# Osimertinib activates a TGF-**β**2–dependent secretory program that drives lung adenocarcinoma progression

**DOI:** 10.1172/JCI198418

**Published:** 2025-12-09

**Authors:** Madhurima Ghosh, Chao Wu, Abhishek Kumar, Monique Nilsson, John V. Heymach, Weina Zhao, Jiang Yu, Xin Liu, Na Ding, Shike Wang, Guan-Yu Xiao, Angelo Chen, Kate Grimley, William K. Russell, Chad J. Creighton, Xiaochao Tan, Jonathan M. Kurie

**Affiliations:** 1 Department of Thoracic/Head and Neck Medical Oncology, The University of Texas–MD Anderson Cancer Center, Houston, Texas, USA.; 2 Section of Hematology and Medical Oncology, Department of Medicine, Tulane University School of Medicine, New Orleans, Louisiana, USA.; 3 Tulane Cancer Center, Louisiana Cancer Research Center, New Orleans, Louisiana, USA.; 4 Department of Toxicology and Cancer Biology, The University of Kentucky, Lexington, Kentucky, USA.; 5 Department of Biosciences, Rice University, Houston, Texas, USA.; 6 Department of Chemistry, Texas A&M University, College Station, Texas, USA.; 7 Department of Biochemistry and Molecular Biology, The University of Texas Medical Branch, Galveston, Texas, USA.; 8 Department of Medicine and Dan L Duncan Comprehensive Cancer Center, Baylor College of Medicine, Texas, USA.

**Keywords:** Cell biology, Clinical Research, Oncology, Cancer, Drug therapy, Oncogenes

## Abstract

EGFR-mutant lung adenocarcinomas (LUADs) that are vulnerable to the EGFR antagonist osimertinib (Osi) eventually relapse, owing in part to the emergence of drug-tolerant persister (DTP) cells that arise through epigenetic mechanisms. Intratumoral DTP cells can herald a worse clinical outcome, but the way in which DTP cells influence LUAD progression remains unclear. Osi-resistant (OR) cells exhibit typical DTP cell features, including a propensity to undergo senescence and epithelial-mesenchymal transition (EMT), which can activate heightened secretory states. Therefore, we postulated that OR cells influence LUAD progression through paracrine mechanisms. To test this hypothesis, we utilized congenic pairs of EGFR-mutant LUAD cell lines in which drug-naive (DN) cells were rendered OR by chronic exposure to escalating doses of Osi. Cocultured in vitro or coinjected into mice, paracrine signals from OR cells enhanced the growth and metastatic properties of DN cells. EMT and senescence activated nonoverlapping secretomes, and OR cells governed DN cells by undergoing EMT but not senescence. Mechanistically, Osi rapidly increased TGF-β2 levels to initiate EMT, which triggered a Golgi remodeling process that accelerated the biogenesis and anterograde trafficking of secretory vesicles. The protumorigenic activity of OR cells was diminished by depletion of EMT-dependent secreted proteins or the EMT-activating transcription factor ZEB1. These findings identify paracrine mechanisms by which OR cells drive LUAD progression.

## Abstract 2

Osimertinib resistant cells secrete effector proteins which through paracrine mechanisms increase the metastatic properties of drug naive cells and influence lung cancer progression.

## Introduction

The term “oncogene addiction” was first coined to describe the reliance of cancer cells on a single mutated oncoprotein or pathway to maintain tumorigenicity (1). These driver mutations confer a growth advantage to cancer cells and form a rationale for the development of molecular targeted therapy (2). In 10%–20% of patients with non–small cell lung cancer, activating mutations in *EGFR* serve as the driver mutation (3, 4). The two most frequently occurring “classical” EGFR mutations, exon 19 deletion mutations and L858R substitution mutations, constitute 85% of observed cases (3, 5). EGFR-selective tyrosine kinase inhibitors (TKIs) that reversibly bind to the ATP-docking site lead to rapid reductions in tumor bulk for patients with lung adenocarcinoma (LUAD) harboring classical EGFR mutations (6). These findings support ongoing efforts to develop targeted therapies based on the oncogene addiction hypothesis.

Despite clear evidence of a survival benefit from reversible EGFR TKIs, tumors eventually regrow in patients who are on these drugs (7, 8). Acquired resistance can result from secondary EGFR mutations, including the gatekeeper T790M mutation (7, 8). To address acquired resistance to EGFR TKIs, osimertinib (Osi) was developed (9, 10). Osi irreversibly binds to the ATP-binding domain of mutant EGFR proteins (6). Treatment regimens involving Osi have yielded highly effective short-term clinical outcomes, but acquired resistance to Osi eventually occurs and remains a major therapeutic challenge (8, 11). In most cases, acquired resistance to Osi is linked to the emergence of a population of drug-tolerant persister (DTP) cells that arise through epigenetic mechanisms, are slow cycling, possess a stem-like phenotype, and are prone to undergoing senescence and epithelial-mesenchymal transition (EMT) (12–17). DTP cells can either revert to an Osi-sensitive state or progress to a drug-tolerant expanded persister cell that exhibits heightened proliferative activity and can advance to an irreversible Osi-resistant (OR) state (18). DTP cells typically constitute a small fraction of total tumor bulk, but their presence predicts a poor clinical outcome (19, 20). Potentially underlying this observation, senescent tumor cells acquire heightened secretory activity that can drive prometastatic processes in the tumor microenvironment (21–25). However, paracrine signals emanating from DTP cell populations have not been fully elucidated.

Proteins secreted through the conventional pathway are synthesized in the endoplasmic reticulum and then transported to the Golgi where they are sorted and packaged into secretory vesicles that are conveyed along microtubules to the plasma membrane (26). The conventional secretory pathway is constitutively activated in cancer by epigenetic events that accelerate secretory vesicle biogenesis (27–29). For example, senescence activates NF-κB to initiate the senescence-associated secretory phenotype (SASP) (30, 31) and EMT-activating transcription factors upregulate the levels of phosphatidylinositol-4-kinase IIα (PI4K2A) and Rab6A to drive the biogenesis and anterograde trafficking of secretory vesicles (24, 25). Here, we postulated that heightened secretory states in DTP cells result from epigenetic events that underlie DTP cell outgrowth and play key roles in LUAD progression.

## Results

### Secretory processes drive biological differences between DN and OR LUAD cells.

**Figure 1:**
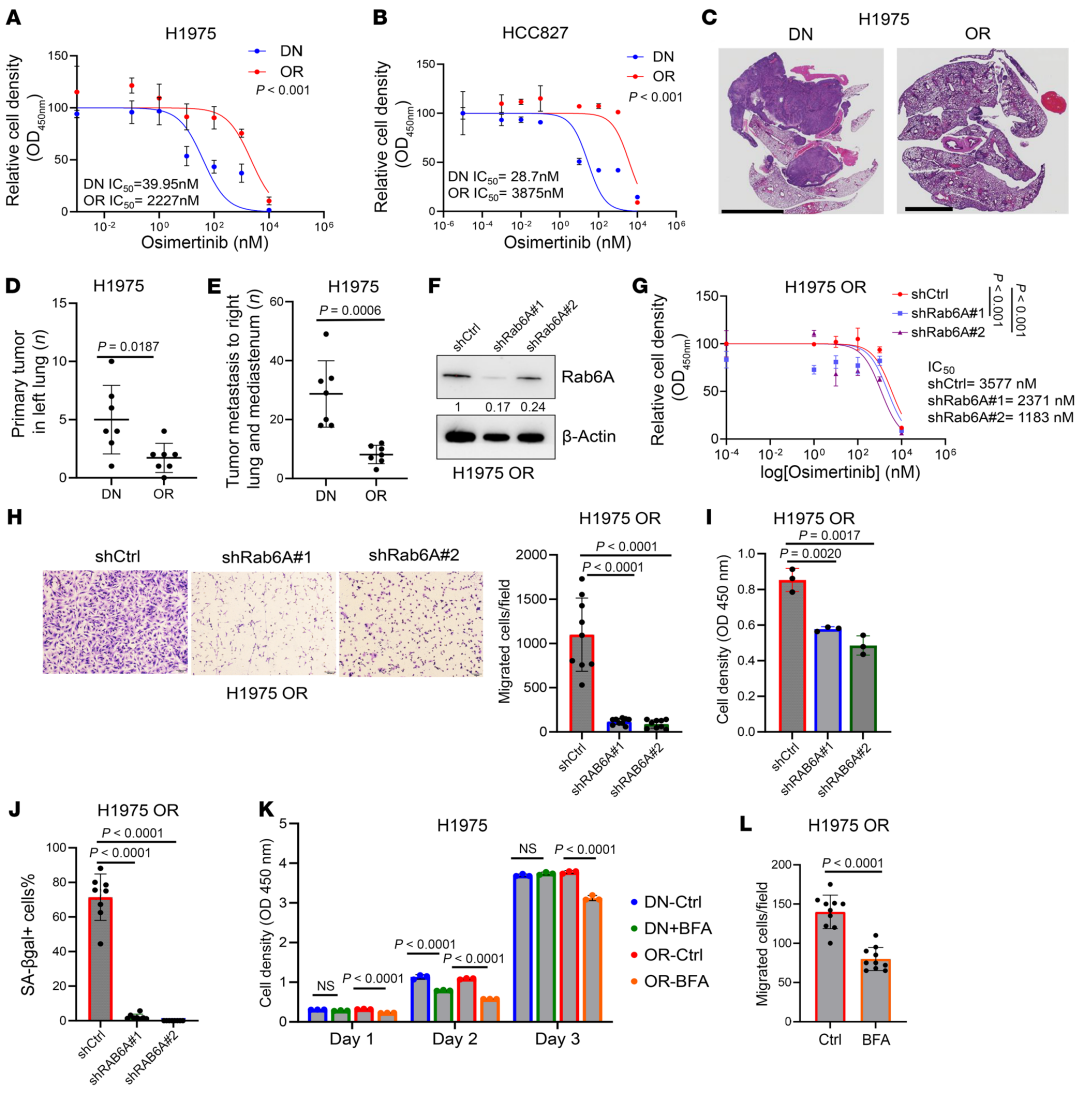
Secretory processes drive biological differences between drug-naive (DN) and Osi-resistant (OR) LUAD cells. (**A** and **B**) Relative densities of H1975 cells (**A**) and HCC827 cells (**B**) (OR and DN) following 3 days of Osi treatment in monolayer culture. Results expressed relative to DMSO control. IC_50_ values (bottom) were calculated. (**C**–**E**) Histology of orthotopic lung tumor sections. Scale bars: 5 mm (**C**). H1975 cells (OR or DN) were injected intrathoracically into *nu*/*nu* mice to grow lung tumors for 6 weeks. At necropsy, each mouse (dot) was scored based on primary lung tumor numbers (**D**) and mediastinal lymph node and contralateral lung tumor metastases (**E**). (**F**) WB confirmation of target gene depletion in shRNA-transfected H1975 OR cells. Densitometric values expressed relative to shCtrl. β-Actin served as loading control. (**G**) Relative densities of shRNA-transfected H1975 OR cells treated with Osi in monolayer culture. IC_50_ values (right) were calculated. (**H**) Boyden chamber migration assays. Scale bars: 100 μm. Results expressed as an average number of migrated cells per field (bar graph). (**I**) Densities of shRNA-transfected cells in monolayer culture. (**J**) Senescence-associated β-galactosidase (SA-βgal) staining assay on shRNA-transfected cells. Results expressed as a percentage of β-galactosidase^+^ cells per field. (**K**) Densities of H1975 cells (OR or DN) in monolayer culture. Cells were pretreated for 4 hours with 10 μg/mL brefeldin A (BFA) and quantified daily for 3 days. (**L**) Boyden chamber migration assays. Cells were pretreated for 4 hours with 10 μg/mL BFA and seeded into chambers. Migrated cells were visualized and quantified 18 hours after seeding. Results expressed as an average number of migrated cells per field. Data are the mean ± SD from a single experiment incorporating biological replicate samples (*n* = 3, unless otherwise indicated) and are representative of at least 2 independent experiments. Two-way ANOVA with Dunnet’s post hoc test (**A**, **B**, and **G**). Two-tailed Student’s *t* test (**D**, **E**, and **L**). One-way ANOVA with Dunnet’s post hoc test (**H**–**K**).

To develop a model for hypothesis testing, we utilized human LUAD cell lines in which EGFR is constitutively activated owing to a deletion mutation (HCC827, ΔE746–A750) or missense point mutations (H1975, L858R/T790M). In monolayer culture, these cells demonstrated low-nanomolar Osi half maximal inhibitory (IC_50_) values (Figure 1, A and B). We treated them with escalating doses of Osi and found that, after 8 weeks of exposure, the emergent cells exhibited sharply higher Osi IC_50_ values (Figure 1, A and B). For the purposes of this study, the emergent cells are designated as “OR” cells. The OR cells exhibited no evidence of secondary EGFR mutations by genomic sequencing (Supplemental Figure 1, A and B; supplemental material available online with this article; https://doi.org/10.1172/JCI198418DS1) or EGFR reactivation by Western blot (WB) of EGFR p-Tyr (Y1068) (Supplemental Figure 1, C and D), excluding oncogene reactivation as a basis for acquired resistance. Compared with drug-naive (DN) cells, which were cuboidal and grew in clusters, OR cells were more dispersed and exhibited mesenchymal cell morphologies (Supplemental Figure 1, E–G). Upon Osi withdrawal, OR cells partially regained sensitivity to Osi and/or underwent mesenchymal-epithelial transition (Supplemental Figure 1, H–J), a plasticity consistent with a DTP cell phenotype. Thus, the congenic cell line pairs provided a model to elucidate epigenetic mechanisms by which Osi exposure causes DN cells to progress to an OR state.

Compared with DN cells, OR cells demonstrated lower proliferative activity in monolayer culture (Supplemental Figure 2, A and B), higher fractions of senescent cells based on β-galactosidase staining (Supplemental Figure 2, C–E), increased migratory and invasive activities in Boyden chambers (Supplemental Figure 2, F–I), and reduced tumorigenicity and metastatic activity in mice (Figure 1, C–E). The paradoxical finding that OR cells were more invasive but less metastatic than DN cells is consistent with prior reports (32) and raised the possibility that heterotypic cell-cell interactions govern OR cells in vivo. To test our hypothesis that heightened secretion underlies biologic features of OR cells, we asked whether secretory blockade is sufficient to reverse those features. In OR cells, short hairpin RNA–mediated (shRNA-mediated) depletion of Rab6A, a key driver of secretory vesicle biogenesis and transport (33), reduced Osi IC_50_ values, decreased the migratory and proliferative activities of OR cells, and attenuated β-galactosidase^+^ cell fractions (Figure 1, F–J). Furthermore, treatment with Brefeldin A, which induces Golgi dispersal and inhibits secretion (34), reduced the proliferative and migratory activities of OR cells (Figure 1, K and L). Lastly, we carried out conditioned medium (CM) transfer experiments and found that DN cells acquired features of OR cells following treatment with CM samples from OR, but not DN, cells (Supplemental Figure 3, A–F). These features included higher Osi IC_50_ values, reduced proliferative activity, and higher migratory activity (Supplemental Figure 3, A–F). Notably, the fraction of senescent cells only minimally increased in HCC827 DN cells (Supplemental Figure 3G), suggesting that paracrine effectors are not sufficient to confer all DTP cell properties. These findings support the conclusion that secretion underlies certain biologic features of OR cells.

### OR cells exert paracrine effects on DN cells in tumor models.

**Figure 2:**
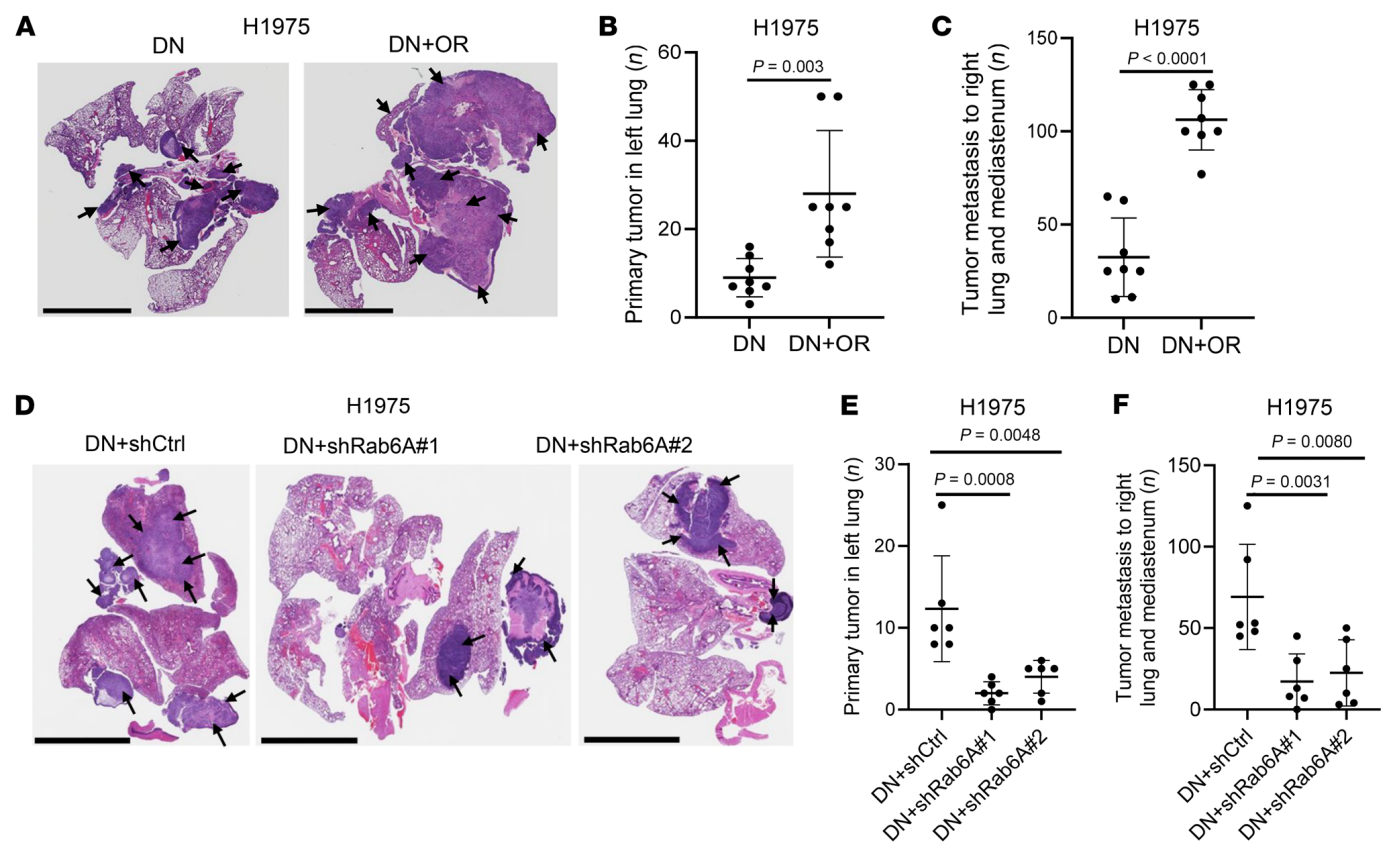
OR cells exert paracrine effects on DN cells in tumor models. (**A**–**C**) Histology of orthotopic lung tumor sections. Scale bars: 5 mm (**A**). DN cells were intrathoracically injected alone (DN) or in combination with OR cells (DN+OR, 10:1 ratio) into *nu*/*nu* mice to grow tumors for 4 weeks. At necropsy, each mouse (dot) was scored as described above (**B** and **C**). (**D**–**F**) Histology of orthotopic lung tumor sections. Scale bars: 5 mm (**D**). DN cells were injected alone or in combination with either Rab6A-replete (shCtrl) or -deficient (shRab6A 1 or 2) OR cells (10:1 ratio) into *nu*/*nu* mice and scored at necropsy (**E** and **F**). Data are the mean ± SD from a single experiment incorporating biological replicate samples (*n* = 3, unless otherwise indicated) and are representative of at least 2 independent experiments. Two-tailed Student’s *t* test (**B** and **C**). One-way ANOVA with Dunnet’s post hoc test (**E** and **F**).

In tumor samples derived from patients and preclinical models, the presence of DTP cells negatively impacts clinical outcome, yet DTP cells constitute a small fraction of total tumor bulk (19, 35). Based on the secretory phenotype exhibited by OR cells, we reasoned that paracrine signals from OR cells may influence the metastatic fate of surrounding tumor cells. To address this possibility, we generated tumors containing DN cells alone or a mixture of DN cells and OR cells (10:1 ratio) and found that tumors generated by OR/DN cell mixtures were larger and more metastatic (Figure 2, A–C). To assess the extent to which paracrine effectors underlie this observation, we compared tumors generated by DN cells mixed with Rab6A-deficient or -replete OR cells and found that mixed-cell tumors generated by Rab6A-deficient OR cells were smaller and less metastatic (Figure 2, D–F). These findings suggest that paracrine signals underlie the capacity of OR cells to drive LUAD progression.

**Figure 3:**
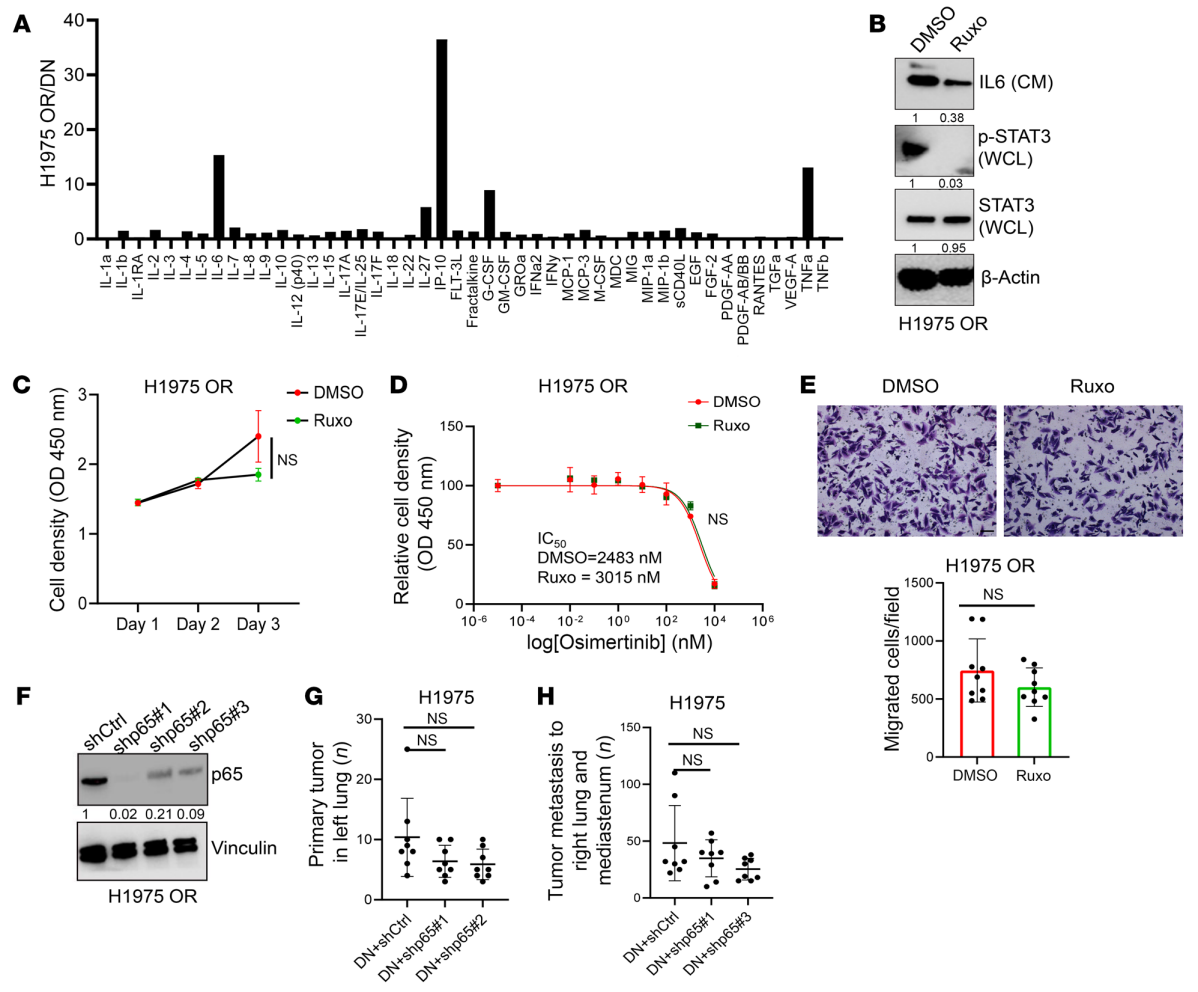
Senescence-activated cytokine secretion in H1975 OR cells. (**A**) Fold change in cytokine concentrations in conditioned medium (CM) samples quantified by multiplexed antibody-based bead assays. Results expressed as a ratio (OR/DN). (**B**) Western blot (WB) analysis of H1975 OR cells treated with the Janus kinase inhibitor ruxolitinib (Ruxo, 0.5 μM) to inhibit senescence-activated secretion. p-STAT3 levels in whole-cell lysate (WCL) included as positive control for drug target inhibition versus vehicle (DMSO) control. (**C** and **D**) Relative densities of H1975 OR cells treated with Ruxo alone (**C**) or in combination with varying doses of Osi (**D**). (**E**) Boyden chamber migration assays on H1975 OR cells treated with Ruxo. Results expressed as an average number of migrated cells per field. Scale bars: 1 mm. (**F**) WB analysis confirming target gene depletion in shRNA-transfected H1975 OR cells. shCtrl, control. Vinculin served as loading control. (**G** and **H**) DN cells were injected in combination with cells described in **F** (10:1 ratio) into *nu*/*nu* mice. At necropsy, each mouse (dot) was scored based on primary lung tumor numbers (**G**) and mediastinal lymph node and contralateral lung tumor metastases (**H**). Data are the mean ± SD from a single experiment incorporating biological replicate samples (*n* = 3, unless otherwise indicated) and are representative of at least 2 independent experiments. Two-way ANOVA with Dunnet’s post hoc test (**C** and **D**). Two-tailed Student’s *t* test (**E**). One-way ANOVA with Dunnet’s post hoc test (**G** and **H**).

Based on our finding that a fraction of OR cells were β-galactosidase^+^, we reasoned that SASP may underlie the biologic differences between OR cells and DN cells. However, quantification of 48 different cytokines known to be SASP associated showed that only 4 of them (IL-6, TNF-α, IP-10, and G-CSF) were present at higher concentrations in CM samples from OR cells than DN cells (Figure 3A). Because IL-6 and TNF-α can drive EMT and drug resistance (36, 37), we utilized genetic and pharmacologic approaches to block SASP (38, 39). Treatment with the JAK inhibitor ruxolitinib (39) to block SASP reduced the levels of those secreted proteins but had no detectable effect on the proliferative or migratory properties of OR cells (Figure 3, B–E). Similarly, mixed-cell tumors generated by DN cells and OR cells that were either NF-κB deficient or replete were similar in size and metastatic activity (Figure 3, F–H). Although these findings do not completely exclude a role for cytokine secretion, they suggest that SASP was not a major contributor to the biological properties of OR cells under these conditions.

### Secretory mediators in OR cells.

**Figure 4:**
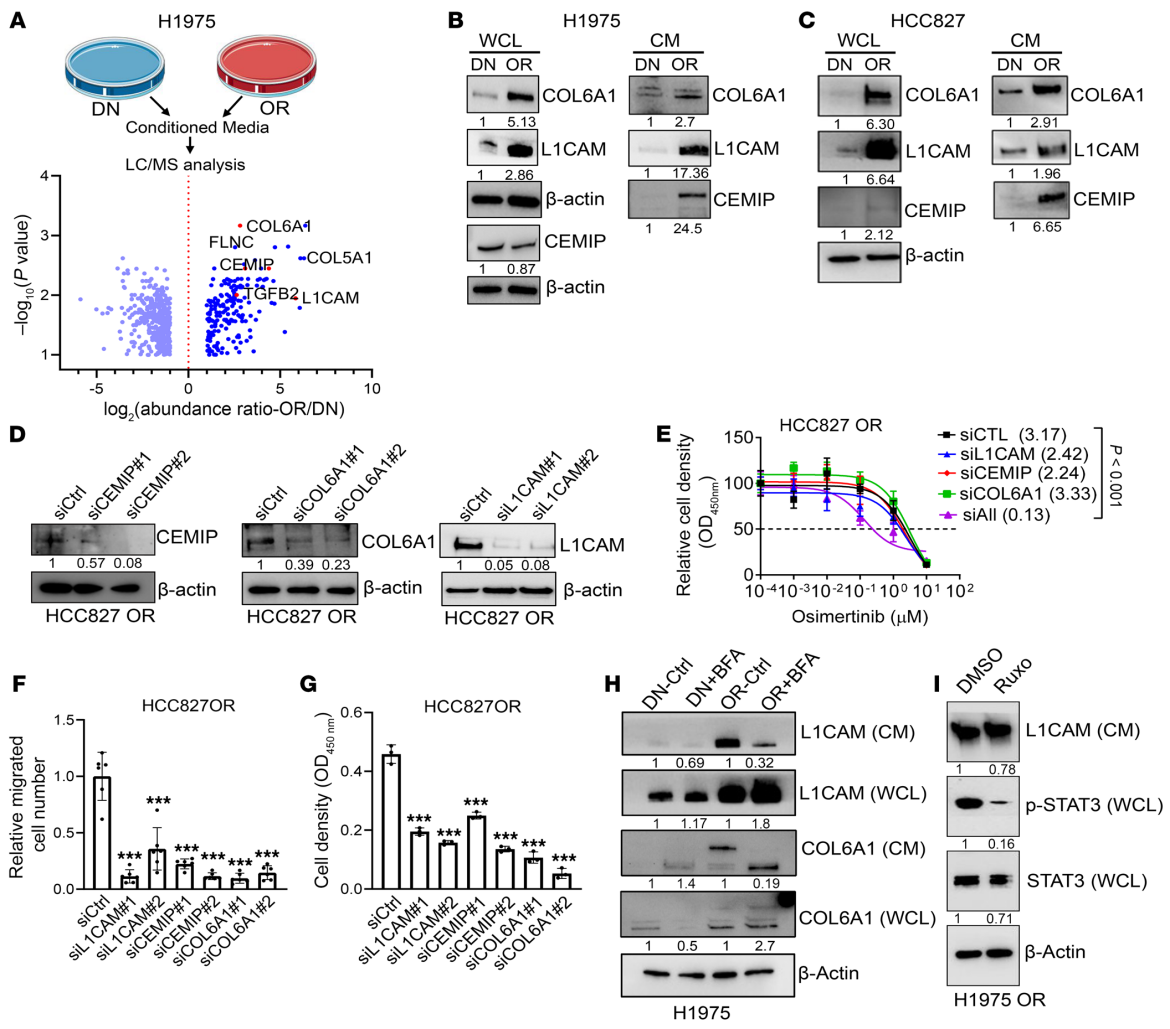
Identification of secretory mediators in H1975 OR cells. (**A**) Volcano plot of proteins identified by LC-MS analysis of CM samples isolated from H1975 cells (DN or OR). Proteins (dots) plotted by *P* value (*y* axis) and fold change (*x* axis). Fold change > 2, *P* < 0.05. Proteins of interest are labeled. (**B** and **C**) WB analysis of secreted proteins of interest in whole-cell lysate (WCL) and CM samples from H1975 cells (DN or OR) (**B**) or HCC827 cells (DN or OR) (**C**). β-Actin served as loading control. (**D**) WB confirmation of target gene depletion (CEMIP, COL6A1, or L1CAM) in siRNA-transfected HCC827 cells. siCtrl, control. β-Actin served as loading control. (**E**) Relative densities of siRNA-transfected HCC827 OR cells following 4 days of Osi treatment in monolayer culture. IC_50_ values were calculated. (**F**) Boyden chamber migration assays on siRNA-transfected cells. Results expressed relative to siCtrl. (**G**) Relative densities of siRNA-transfected HCC827 cells in monolayer culture quantified on day 4. (**H**) WB analysis of L1CAM and COL6A1 in CM and WCL samples following treatment with brefeldin A (10 μg/mL) for 4 hours in H1975 DN and OR cells. (**I**) WB analysis of L1CAM in CM samples from H1975 OR cells after 24-hour treatment with 1 μM Ruxo. p-STAT3 levels in WCL included as positive control for drug target inhibition. Data are the mean ± SD from a single experiment incorporating biological replicate samples (*n* = 3, unless otherwise indicated) and are representative of at least 2 independent experiments. Two-way ANOVA with Dunnet’s post hoc test (**E**). One-way ANOVA with Dunnet’s post hoc test (**F** and **G**). ***P < 0.001.

To more broadly assess the OR cell–derived secretome, we carried out liquid chromatography–mass spectrometry (LC-MS) on CM samples isolated from H1975-matched DN and OR cells. We identified a total of 580 proteins, 152 (26%) of which were present at markedly higher concentrations (*P* < 0.05, >2-fold change) in CM samples from OR cells (Figure 4A). Gene Ontology analysis of the OR cell secretome revealed enrichment in terms such as “regulation of cellular response to growth factor stimulus,” “regulation of cytokine production,” “inflammatory response,” “cell adhesion,” “blood vessel morphogenesis,” and “extracellular matrix organization” (Supplemental Figure 4A). Among the upregulated proteins, collagen VI A1 (COL6A1), L1 cell adhesion molecule (L1CAM), and cell migration–inducing hyaluronidase (CEMIP) were of particular interest based on their correlations with shorter survival durations in The Cancer Genome Atlas (TCGA) LUAD cohort (Supplemental Figure 4, B–D) and reports that they exert protumorigenic activities (40–42). COL6A1 is a component of the triple helical COL6 molecule (α1/α2/α3), L1CAM is a transmembrane protein involved in cell adhesion, and CEMIP is an extracellular matrix protein that binds hyaluronidase and induces hyaluronic acid depolymerization (43–45). By WB analysis, the levels of all 3 proteins were higher in CM samples from OR cells than DN cells (Figure 4, B and C), and WB analysis of cell lysates demonstrated a similar pattern for L1CAM and COL6A1 but not CEMIP (Figure 4, B and C). Based on findings from small interfering RNA–mediated (siRNA-mediated) depletion studies, secreted L1CAM, COL6A1, and CEMIP were mediators of Osi resistance (Figure 4, D and E, and Supplemental Figure 4E) and enhanced the proliferative and migratory activities of OR cells (Figure 4, F and G, and Supplemental Figure 4, F–I). Treatment of OR cells with Brefeldin A decreased the secretion of L1CAM and COL6A1, whereas treatment with a JAK inhibitor failed to do so (Figure 4, H and I). These findings suggested that OR cells acquire biologic properties through a secretory program that is SASP independent.

### Conventional secretory pathway activation in OR cells.

We sought to elucidate the mechanistic basis for the heightened secretion in OR cells. Fluorescently tagged Rab6A^+^ secretory vesicles were more numerous in OR cells than DN cells (Supplemental Figure 5, A and B) and a fluorescently tagged version of the temperature-sensitive mutant secretory cargo vesicular stomatitis virus-G (VSV-G) was transported from the endoplasmic reticulum to plasma membrane via the Golgi at a faster rate in OR cells than DN cells (Supplemental Figure 5, C and D), suggesting that the conventional secretory pathway is activated in OR cells. LC-MS analysis of Golgi-fractionated and vesicular subfractionated proteins isolated from H1975-matched DN and OR cells (Supplemental Figure 5E) identified a total of 522 proteins, 271 of which were present at higher levels (*P* < 0.05, >2-fold change) in OR cells (Supplemental Figure 5, F and G), including multiple effectors of the conventional secretory pathway such as RAB GTPases (RAB6C, RAB11B, RAB32) and their associated regulators (RABGEF1, ARHGEF1, ARHGEF2), a molecular motor protein (KIF3b), and a clathrin assembly protein (PICALM). WB analysis of Golgi fractions and vesicle-enriched subfractions confirmed these findings (Supplemental Figure 5H). Thus, OR cells and DN cells demonstrated proteomic differences that might underlie heightened secretion in OR cells.

**Figure 5:**
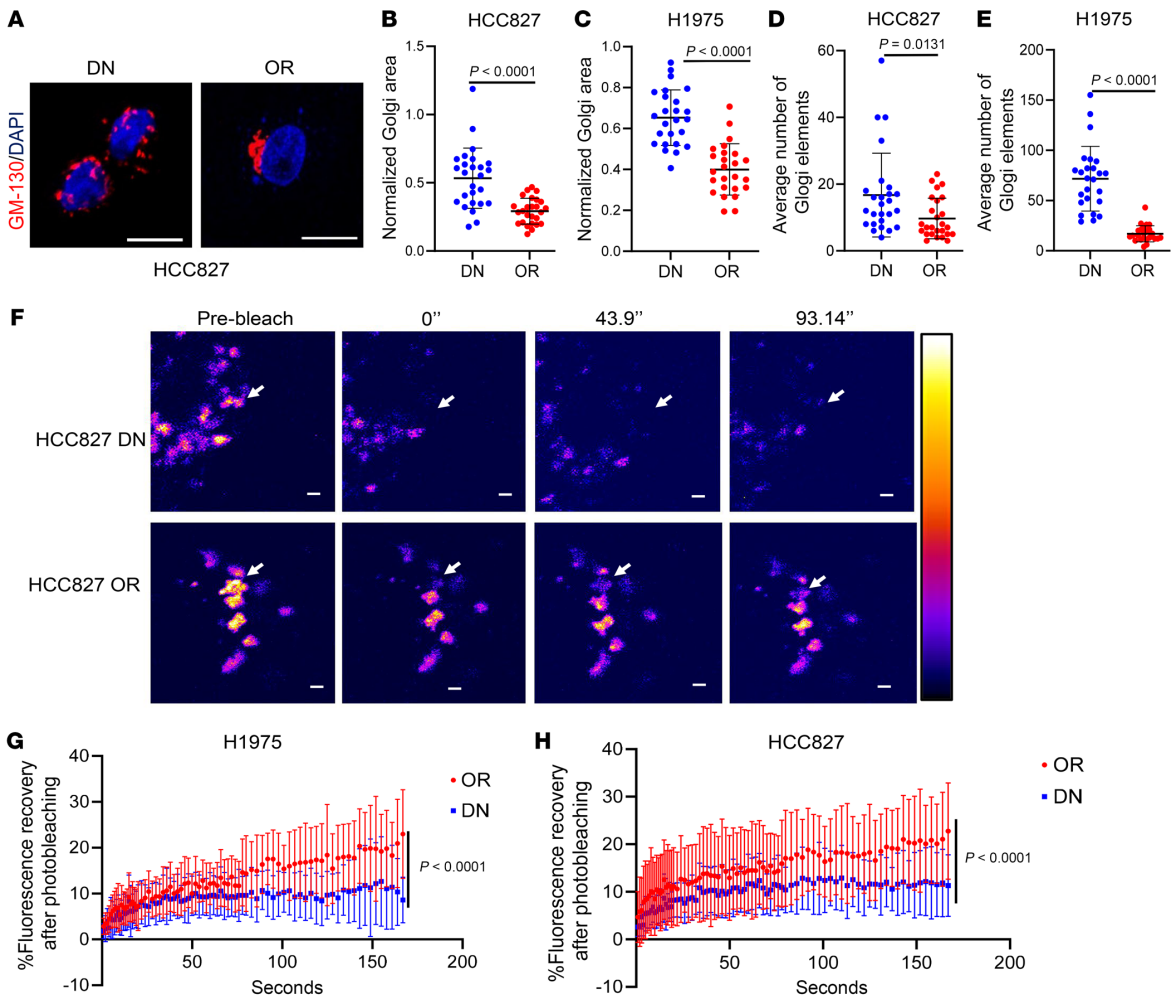
Distinct Golgi dynamics in OR cells and DN cells. (**A**) Confocal micrographs of anti-GM130 antibody–stained HCC827 cells (DN and OR). Golgi (red). Nuclei (DAPI, blue). Scale bars: 10 μm. (**B** and **C**) Scatter plots of Golgi area per cell (dot) in HCC827 cells (**B**) and H1975 cells (**C**). Values were normalized to nucleus area. (**D** and **E**) Scatter plots of Golgi element numbers per cell (dot) in HCC827 cells (**D**) and H1975 cells (**E**). (**F**) Fluorescence recovery after photobleaching (FRAP) assays. Pseudocolored images of the Golgi enzyme N-acetylglucosaminyltransferase at indicated time points after photobleaching. Bleached regions of interest (arrows). Intensity levels are indicated by a lookup (LUT) table bar (right). Scale bars: 1 μm. (**G** and **H**) Scatter plot of intensity recovery after photobleaching (%) in H1975 cells (**G**) and HCC827 cells (**H**). Each dot represents a mean value from *n* = 14 cells per group. Data are the mean ± SD from a single experiment and are representative of at least 2 independent experiments. Two-tailed Student’s *t* test (**B**–**E**). Two-way ANOVA with Dunnet’s post hoc test (**G** and **H**).

A key regulator of the conventional secretory pathway, the Golgi is composed of flattened membranous cisternae that can disperse or condense and form intercisternal, membrane-to-membrane bridges that generate cisternal stacks (46). Cargos diffuse across intercisternal bridges and thereby travel from the *cis* to the *trans* face of the Golgi stack (47). To assess the extent to which the OR cell phenotype is associated with Golgi structural changes, we quantified Golgi organellar areas and Golgi element numbers and sizes (48), which showed that, compared with DN cells, OR cells generate smaller Golgi in which Golgi elements were larger and fewer in number, indicative of a Golgi compaction process (Figure 5, A–E). To analyze intercisternal connectivity, we performed fluorescence recovery after photobleaching (FRAP) assays using a GFP-tagged Golgi enzyme N-acetylglucosaminyltransferase, which showed that, relative to DN cells, OR cells had faster recovery (Figure 5, F–H), an indication of greater intercisternal connectivity.

### EMT drives secretion in OR cells.

**Figure 6:**
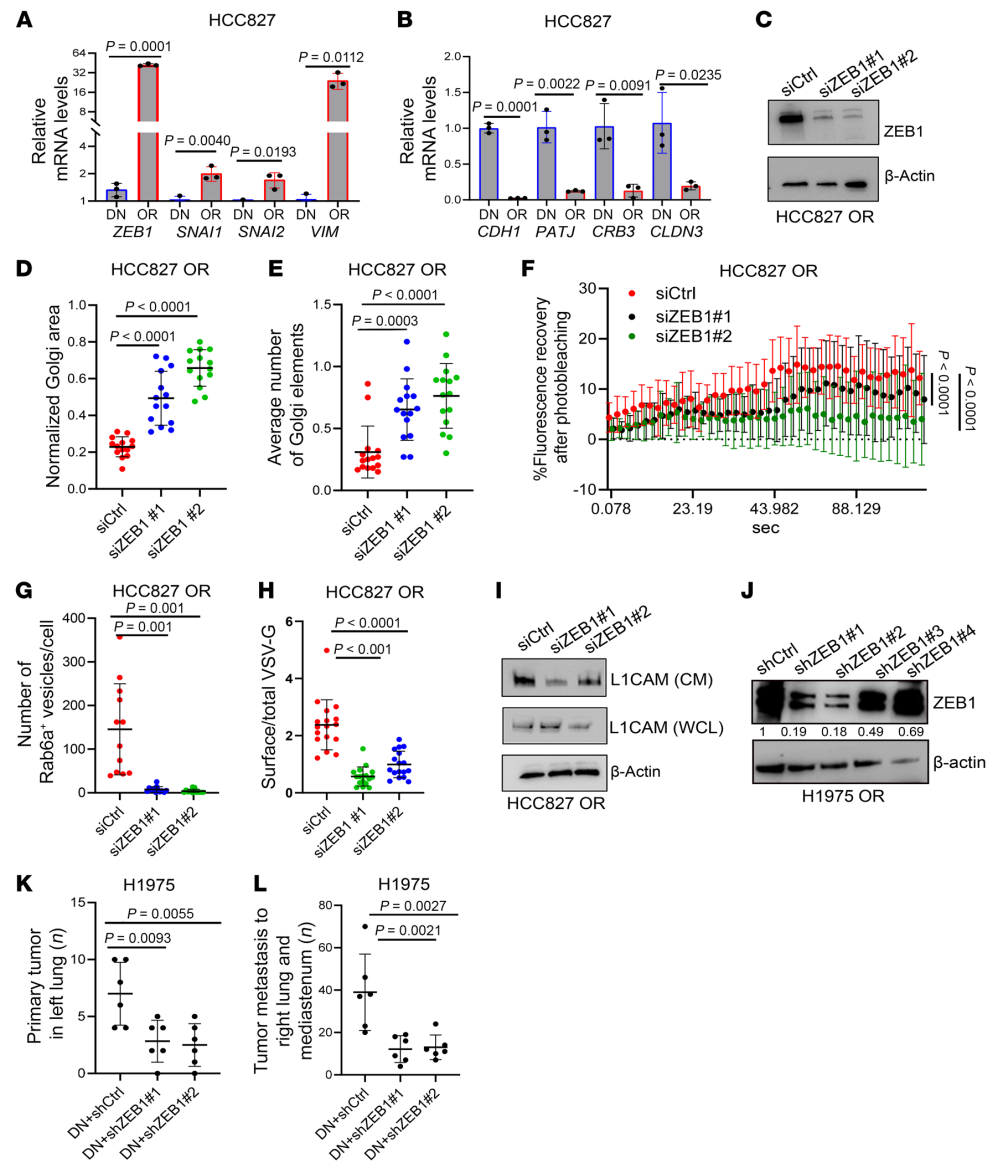
EMT drives Golgi and secretory dynamics in OR cells. (**A** and **B**) Quantitative PCR analysis of mRNA levels in HCC827 cells (OR and DN). Relative levels of EMT-activating transcription factors (ZEB1, SNA1, SNAI2), and mesenchymal marker VIM (**A**). Relative levels of epithelial polarity complex components (CDH1, PATJ, CRB3, and CLDN3) are indicative of EMT in OR cells (**B**). (**C**) WB confirmation of siRNA-mediated target gene depletion in HCC827 OR cells. β-Actin served as loading control. (**D**) Scatter plot of Golgi areas per siRNA-transfected cell (dot). Values normalized to nucleus area. (**E**) Scatter plot of Golgi element numbers per cell (dot). (**F**) Scatter plot of intensity recovery (%) after photobleaching (*n* = 10 cells per group). (**G**) Quantification of Rab6A^+^ vesicles per cell (dot). Vesicles identified based on GFP-tagged Rab6A reporter activity. (**H**) Ratio of surface-to-total VSV-G per cell (dot) infected with adenovirus expressing EGFP-VSV-G and imaged 30 minutes after transfer to 32°C. (**I**) WB analysis of L1CAM in CM and whole-cell lysate samples. (**J**) WB confirmation of shRNA-mediated target gene depletion in H1975 OR cells. β-Actin served as loading control. (**K** and **L**) DN cells were injected in combination with cells described in **J** (10:1 ratio) into *nu*/*nu* mice. At necropsy, each mouse (dot) was scored based on primary lung tumor numbers (**K**) and mediastinal lymph node and contralateral lung tumor metastases (**L**). Data are the mean ± SD from a single experiment incorporating biological replicate samples (*n* = 3, unless otherwise indicated) and are representative of at least 2 independent experiments. Two-tailed Student’s *t* test (**A** and **B**). One-way ANOVA with Dunnet’s post hoc test (**D**, **E**, **G**, **H**, **K**, and **L**). Two-way ANOVA with Dunnet’s post hoc test (**F**).

Golgi compaction, enhanced intercisternal connectivity, and heightened secretion are components of an integrated, EMT-driven process (48). OR cells exhibited morphologic and transcriptional evidence of EMT (Figure 6, A and B, Supplemental Figure 1C, and Supplemental Figure 6, A and B), including higher levels of transcriptional drivers of EMT (SNAI1, ZEB1). In TCGA LUAD cohort, proteins upregulated in the OR secretome were correlated with an EMT gene expression signature (Supplemental Figure 6C). Therefore, we reasoned that EMT may be a driver of heightened secretion in OR cells and addressed this possibility by carrying out ZEB1 gain- and loss-of-function studies on DN cells and OR cells, respectively, which showed that ZEB1 enhanced the biogenesis and anterograde transport of secretory vesicles (Figure 6, C–H, and Supplemental Figure 6, D and E), increased L1CAM secretion (Figure 6I and Supplemental Figure 6F), augmented Osi resistance and mesenchymal features (Supplemental Figure 6, G–J), and mediated paracrine effects of OR cells on DN cells in mice (Figure 6, J–L). Thus, EMT underlies heightened secretion in OR cells.

### Osi activates TGF-β2 secretion to initiate the EMT-activated secretory pathway.

**Figure 7:**
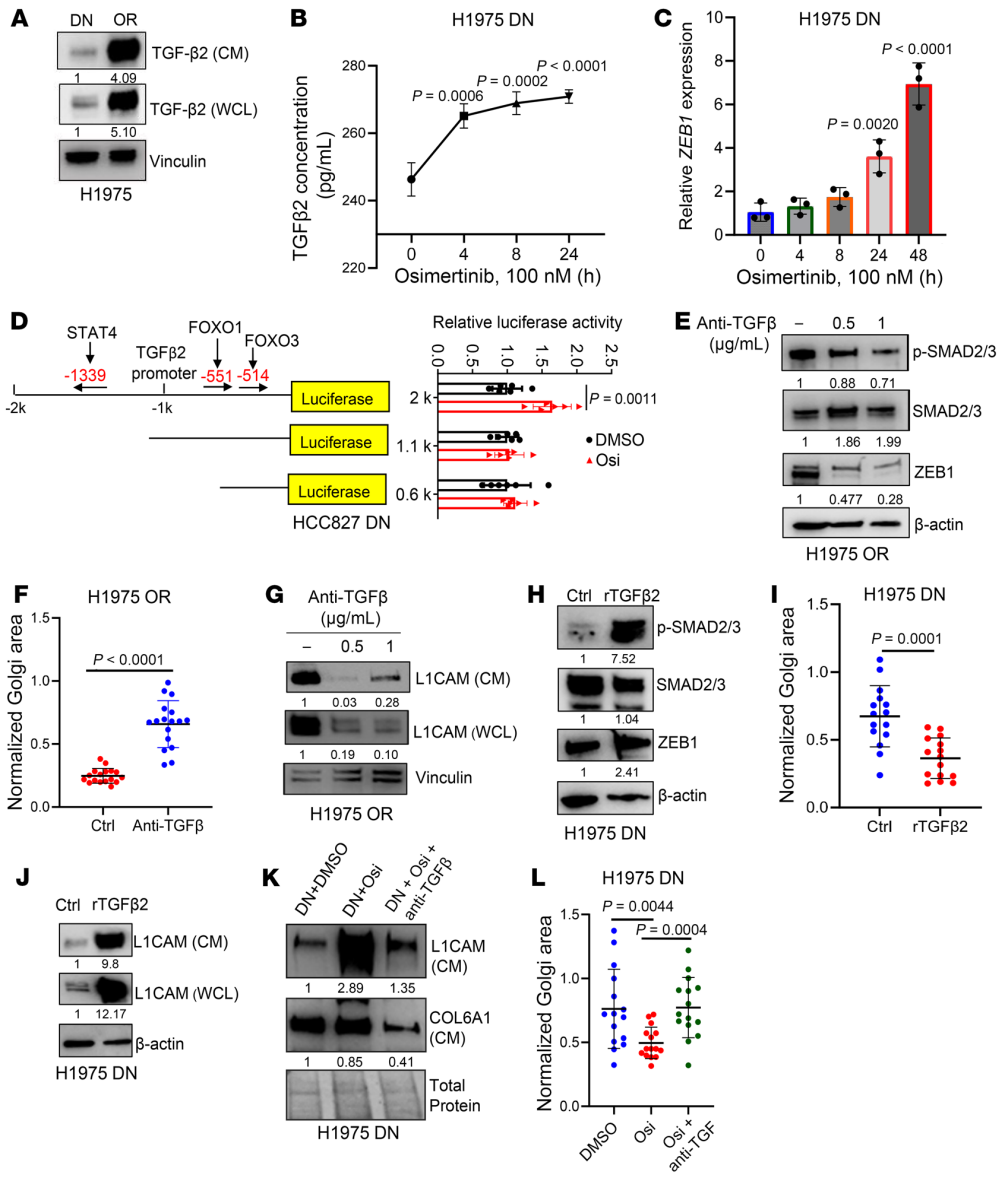
Osimertinib activates TGF-β2 secretion to initiate the EMT-dependent secretory pathway. (**A**) TGF-β2 WB analysis of CM and whole-cell lysate (WCL) samples from H1975 cells (DN or OR). (**B**) Enzyme-linked immunosorbent assays of CM samples show that increased TGF-β2 secretion is detectable 4 hours after initiating Osi treatment in H1975 DN cells. (**C**) Quantitative PCR analysis shows that ZEB1 mRNA levels are upregulated 24 hours after initiating Osi treatment in H1975 DN cells. (**D**) TGF-β2 gene promoter reporter assays. Reporters containing a 2-kb TGF-β2 gene promoter fragment or truncated (1.1 kb or 0.6 kb) fragments were transfected into HCC827 DN cells. After 48 hours, cells were treated with DMSO or 100 nM Osi for 24 hours. Values were normalized to *Renilla*, averaged from replicates (*n* = 6), and expressed relative to DMSO. Predicted transcription factor binding sites shown in schematic (left). (**E**) WB confirmation of SMAD2/3 dephosphorylation by 0.5 and 1 μg/mL neutralizing anti–TGF-β antibody treatment of H1975 OR cells. β-Actin served as loading control. (**F**) Scatter plot of Golgi areas following TGF-β neutralization in H1975 OR cells (dots). Values normalized based on nucleus area. (**G**) WB analysis of L1CAM in CM and WCL samples following TGF-β neutralization in H1975 OR cells. (**H**) WB confirmation of increased SMAD2/3 phosphorylation by recombinant TGF-β2 (100 nM) treatment of H1975 DN cells. β-Actin served as loading control. (**I**) Golgi areas following recombinant TGF-β2 treatment in H1975 DN cells (dots). Values normalized based on nucleus area. (**J**) WB analysis of L1CAM in CM and WCL samples following recombinant TGF-β2 treatment of H1975 DN cells. (**K**) WB analysis of L1CAM and COL6A1 in CM samples from H1975 DN cells treated for 24 hours with DMSO, 100 nM Osi, or Osi in combination with 1 μg/mL neutralizing anti–TGF-β antibody. Ponceau-stained gel included as loading control. (**L**) Scatter plot of Golgi areas in H1975 DN cells (dots) treated with Osi alone or in combination with neutralizing anti–TGF-β antibody. Data are the mean ± SD from a single experiment incorporating biological replicate samples (*n* = 3, unless otherwise indicated) and are representative of at least 2 independent experiments. One-way ANOVA with Dunnet’s post hoc test (**B**, **C**, and **L**). Two-tailed Student’s *t* test (**D**, **F**, and **I**).

While EMT is a common feature of OR cells (49), the signals that induce EMT in OR cells remain unclear. TGF-β is a potent EMT inducer, and its secretion is reportedly enhanced upon treatment of LUAD cells with an EGFR antagonist (50, 51). In line with this observation, TGF-β2 levels were higher in CM samples from OR cells than DN cells (Figure 3A, Figure 7A, and Supplemental Figure 7A). In DN cells, TGF-β2 secretion increased within 4 hours of Osi treatment initiation, and the TGF-β2 increase preceded ZEB1 mRNA upregulation (Figure 7, B and C, and Supplemental Figure 7, B and C). In DN cells, Osi treatment activated a TGF-β2 promoter reporter, and serial deletion studies identified a promoter region that mediated the Osi-induced activation (Figure 7D). Treatment of OR cells with anti–TGF-β2 neutralizing antibodies decreased ZEB1 expression (Figure 7E and Supplemental Figure 7D), dispersed the Golgi (Figure 7F and Supplemental Figure 7E), and reduced L1CAM secretion (Figure 7G and Supplemental Figure 7F). In DN cells, treatment with recombinant TGF-β2 increased ZEB1 expression (Figure 7H and Supplemental Figure 7G), induced Golgi compaction (Figure 7I and Supplemental Figure 7H), and heightened L1CAM secretion (Figure 7J and Supplemental Figure 7I). TGF-β2–neutralizing antibodies reversed the effect of Osi on these parameters (Figure 7, K and L, and Supplemental Figure 7, J–L). In contrast, ruxolitinib treatment to block SASP had no detectable effect on Golgi areas in OR cells (Supplemental Figure 7M). We conclude that TGF-β2 secretion activates an EMT-dependent secretory process that drives biologic properties of OR cells.

## Discussion

The “tumor as organizer” model establishes the cancer cell as the primary architect of the tumor microenvironment (21). The cancer cell secretome is comprised of soluble proteins and insoluble vesicles that, through autocrine and paracrine mechanisms, sustain cancer cell survival and recruit various cell types to create an immunosuppressive and fibrotic tumor stroma (52). Continuous exposure to anticancer drugs can alter the tumor cell secretome to facilitate drug resistance and disease relapse after therapy (22, 53, 54). Here, we show that Osi treatment of DN cells rapidly activates a heightened secretory state that underlies DTP and OR cell emergence. These findings identify paracrine mechanisms by which OR cells drive LUAD progression.

DTP cells were first identified as bacteria that survive antibiotic treatment through the acquisition of gene mutations (55). In cancer, there is an ongoing debate over whether DTP cells preexist in treatment-naive tumors or evolve from DN cells through induced phenotypic changes (13, 35, 56–58). DTP cells exhibit hallmark features of stem cells, including the capacity to undergo senescence and/or EMT (12, 14, 17, 35). Heightened secretion in DTP cells has been linked to senescence (12, 23), but the findings presented here suggest that senescence is not the sole contributor. We show that Osi induces rapid activation of a TGF-β2/ZEB1–dependent pathway that initiates Golgi remodeling and accelerates the biogenesis and anterograde trafficking of secretory vesicles containing key effectors of the DTP cell phenotype. Thus, we propose that, beyond its established role in promoting Osi resistance in DTP cells (49, 59), EMT activates a secretory program that drives LUAD progression.

DTP-selective therapeutic strategies have been identified that combine Osi with drugs that target unique metabolic features, signaling pathways, or cell surface molecules in DTP cells (12, 17, 49, 60–64). We reported previously that EMT-dependent secretion drives LUAD progression and is therapeutically actionable with selective antagonists of PI4K2A, a Golgi- and endosome-resident enzyme that is upregulated during EMT and drives the biogenesis and trafficking of secretory vesicles and recycling endosomes (25, 65, 66). PI4K2A antagonists are under clinical development for non-oncologic indications (25) and could be repurposed for clinical trials testing their activity against DTP cells in the setting of Osi resistance.

Here we found that Osi activated the secretory program within hours of treatment initiation, suggesting that Osi exerts protumorigenic activity before OR cells emerge. This secretory program could represent a type of non-oncogene addiction (67, 68) if Osi and TGF-β2 inhibition are found to be synthetically lethal in EGFR-mutant LUAD cells. Furthermore, the rapidity with which Osi activated secretion in DN cells raises the possibility that secretory blockade could delay the outgrowth of resistant cells and thereby enhance the therapeutic activity of Osi in DN LUAD patients. This finding could have potential clinical relevance in the front-line treatment setting by utilizing TGF-β antagonists. Indeed, TGF-β2 is a recognized contributor to Osi resistance in LUAD cells and could be targeted with anti–TGF-β2 neutralizing antibodies that are under clinical development (69–71).

Several shortcomings in our study warrant discussion. First, while these findings identify an EMT-activated secretory process that is distinct from SASP, whether the EMT- and senescence-activated secretory programs are activated in overlapping or distinct OR cell populations remains unclear. Indeed, senescence may not be strictly limited to DTP cells but may also occur in a totally separate population of OR cells (12, 72, 73). Addressing this question has clinical ramifications because senescence is a hallmark on which current efforts to therapeutically target DTP cells is based (74). Second, secretory blockade inhibited certain DTP cell features more prominently than others. For example, proliferation and migration were attenuated to a greater extent than Osi resistance was reversed, suggesting that those features are governed independently. The mechanistic basis for this observation remains unclear. Third, whether the secretory program we identified in cell culture models is operative in patients remains unclear. Elucidating the extent to which TGF-β2 underlies Osi resistance in LUAD patients is an important question and must be addressed before TGF-β2 targeting is incorporated into Osi-based therapeutic strategies.

## Methods

### Sex as a biological variable.

Our study examined male *nu*/*nu* mice because male animals exhibited less variability in phenotype. It is not known whether these findings are relevant for female mice.

### Cell lines.

Congenic pairs of human EGFR-mutant LUAD cell lines (H1975, HCC827) that are DN or OR were obtained in-house and were described previously (49). HCC827_Vec and HCC827_ZEB1 cell lines have been described previously (48). Human LUAD cell lines were cultured in RPMI-1640 supplemented with 10% FBS and were maintained at 37°C in an incubator with a humidified atmosphere containing 5% CO_2_. Cells were transfected using XtremeGene 9 DNA Transfection Reagent (Roche) and stable transfectants were selected after 2 weeks following puromycin treatment. The OR cells were maintained in 10% FBS media with 1 μM Osi. Cells were routinely tested for mycoplasma infections.

### Reagents.

We purchased FBS, RPMI-1640, live-cell imaging solution, PBS, Alexa Fluor–tagged secondary antibodies, paraformaldehyde, BSA, DAPI, Triton X-100, Lipofectamine RNAiMAX Reagent, and Cell-Light Golgi GFP from Thermo Fisher Scientific; XtremeGene9 DNA transfection reagent was from MilliporeSigma; puromycin from InvivoGene; Transwells and Matrigel-coated Boyden chambers from BD Biosciences; collagen-coated glass-bottom dishes and multiwell plates from MatTek; 8-well chamber slides from Thermo Fisher Scientific; trypsin from Corning; qScript cDNA super-Mix from Quanta Biosciences; SYBR Green Real-Time PCR Master Mixes from Bimake; Fast Mix from Quanta Biosciences; 10× cell lysis buffer and protease/phosphatase inhibitor cocktail from Cell Signaling Technology; CCK-8 (catalog K1018) from APExBIO; Brefeldin A (catalog B7651) from Sigma-Aldrich; human recombinant TGF-β2 protein (catalog 100-35B) from Peprotech; anti-DCTN1 (catalog MAB6657-SP) and anti-TGFβ1/-2/-3 antibody (catalog MAB1835R-SP) from R&D Systems; Osi (catalog S7297) from Selleckchem; ruxolitinib (catalog 941678-49-5) from MedChem Express; RNeasy Plus Mini Kit (catalog 74136) from QIAGEN; siRNAs against human ZEB1 (catalog SASI_Hs01_0011_1599; SASI_Hs01_0011_1598), L1CAM (catalog SASI_Hs01_00084030; SASI_Hs01_00084031), COL6A1 (catalog SASI_Hs01_00014661; SASI_Hs01_00014662), CEMIP (catalog SASI_Hs01_00241050; SASI_Hs01_00241049), and shRNAs against human Rab6A (catalog TRCN0000047985; TRCN0000047987), ZEB1 (catalog TRCN0000017566;TRCN0000017565), and p65 (catalog TRCN0000014683; TRCN0000014686) from MilliporeSigma; primary antibodies against p-EGFR (catalog 8543S), SMAD (catalog 8685T), p-SMAD (catalog 8828S), vinculin (catalog 4650S), Rab6A (catalog 4879S), GM130 (catalog 12480S), β-actin (catalog 4970S), p65 (catalog 8242T), IL-6 (catalog 12153), p-STAT3 (catalog 9131S), STAT-3 (catalog 9132) from Cell Signaling Technology; primary antibodies against L1CAM (catalog L4543) from MilliporeSigma; primary antibodies against EGFR (catalog sc-03), COL6A1 (catalog sc-377143), ACBD3 (catalog sc-101277), KIF3B (catalog sc-514165) from Santa Cruz Biotechnology; primary antibodies against CEMIP (catalog 21129-1-AP), Myo9B (catalog 12432-1-AP), Rabep1 (catalog 14350-1-AP), Rabgef1 (catalog 12735-1-AP), PICALM (catalog 26765) from Proteintech; primary antibodies against VSV-G (catalog EB0012) from Kerafast; primary antibodies against GEF-H1 (catalog GTX125893) or ZEB1 (catalog GTX105278) from GeneTex; Alexa Fluor 568–phalloidin (catalog A12380) from Thermo Fisher Scientific; WT Rab6A-EGFP expression vector (catalog 49469) from Addgene; and adenovirus expressing EGFP-tagged temperature sensitive mutant VSV-G was provided by Anne Muesch (Albert Einstein College of Medicine, New York, New York, USA).

### Animal experiments.

The nude mice were purchased from The Jackson Laboratory. Mice underwent standard care and were euthanized at predetermined time points or at the first signs of morbidity according to the standards set forth by the Institutional Animal Care and Use Committee. To generate subcutaneous tumors *nu*/*nu* mice (*n* = 5–10 per group) were subcutaneously injected with 1 × 10^6^ human lung cancer cells. To generate orthotopic lung tumors, *nu*/*nu* mice were intrathoracically injected with 1 × 10^6^ human LUAD cells containing DN cells alone or in combination with OR cells (10:1 ratio). Primary lung tumors and lung metastases were measured, counted, and confirmed by H&E staining.

### Vector construction.

The human TGF-β2 promoter fragment containing 1837 bp upstream and 137 bp downstream of the transcription start site (TSS) was amplified from genomic DNA of H1299 cells using the primers 5′-TTTCTCTATCGATAGGTACCGAGCTCGTCTATAATGGCCACAGGTGTAAG-3′ and 5′-AGCTTACTTAGATCGCAGATCTCGAGCCTCTTTCACTTGCGCTCTC-3′. The PCR product was then cloned into the pGL3-Enhancer vector (Promega) using Gibson assembly. Two 5′-truncated TGF-β2 promoter constructs containing 1078 bp (1.1 kb) and 634 bp (0.6 kb) upstream of the TSS were amplified using the forward primers 5′-ATTTCTCTATCGATAGGTACCGAGCTCAGGCCCCATACACAACTGAA-3′ and 5′-ATTTCTCTATCGATAGGTACCGAGCTCGCAGCAAATTATAAAGGTGACCATTC-3′, respectively.

### EGFR sequencing.

Exons 18 to 21 of the human EGFR gene were amplified from cDNAs synthesized from parental and OR H1975 and HCC827 cells using the primers 5′-ATCGGCCTCTTCATGCGAAG-3′ and 5′-CGTAGCTCCAGACATCACTCTG-3′. The purified PCR products were subsequently submitted for Sanger sequencing to examine hotspot mutations in EGFR.

### LC-MS analysis.

To identify differentially secreted proteins, we collected and concentrated CM samples from DN cells and OR cells using Amicon Ultra Centrifugal Filters (Sigma-Aldrich) and analyzed them by LC-MS as previously described (66). To identify proteins in the Golgi- and vesicle-enriched subfractions, the fractions were isolated following the manufacturer’s protocol and the samples were analyzed by LC-MS.

### Isolation of Golgi- and vesicle-enriched subcellular fractions.

As previously described (28), the Minute Golgi Apparatus Enrichment Kit (GO-037, Invent Biotechnologies) was used to enrich cell lysates in Golgi and vesicle fractions, following the manufacturer’s instructions.

### Cell proliferation and dose-response assays.

Cells were seeded on 96-well plates at a density of 2000 cells per well and incubated overnight. For dose-response assays, medium was replaced on the following day with 10% FBS–containing RPMI-1640 and various concentrations of Osi (0–10 μM), and cells were allowed to proliferate for 72 hours. Relative cell density was measured by using the CCK-8 Counting Kit according to the manufacturer’s instructions. Briefly, cells were incubated with culture medium containing the CCK-8 solution for 2 hours at 37°C, and the absorbance was read at 450 nm.

### Cell migration and invasion assays.

As described previously (48), 1 × 10^5^ cells were seeded in the upper wells of Transwell plates (BD Biosciences) for migration assays or Matrigel-coated Boyden chambers (BD Biosciences) for invasion assays. Cells were allowed to migrate toward 10% FBS in the bottom wells. After 16 hours of incubation, migrating or invading cells were stained with 0.1% crystal violet, photographed, and counted.

### VSV-G assay.

The VSV-G transport assay was performed as described previously (48, 66). In brief, cells were infected with adenovirus expressing EGFP-VSV-G (ts045). The cells were then transferred to the restrictive temperature of 40°C for 20 hours and transferred to the permissive temperature of 32°C for 1 hour in the presence of 100 mg/mL cycloheximide and then the cells were fixed. In nonpermeabilized cells, exofacial and total VSV-G were detected by staining with an anti–VSV-G antibody and by measurement of EGFP signal intensity, respectively. VSV-G trafficking to the plasma membrane was measured based on the ratio of exofacial (surface) VSV-G fluorescence signal to the EGFP (total) signal intensity.

### CM transfer assay.

Cells (2 × 10^6^) were plated in 10-cm dishes and incubated overnight. The cells were serum starved for 16 hours and the supernatants (CM) were collected, filtered through a 0.45-μm filter, mixed with complete growth medium (1:1), and added to cells. Cells were pretreated with respective CM samples for 3 days, following which the cells were trypsinized and plated for further assays.

### β-Galactosidase staining assay.

Cells were plated in 35-mm dishes and following predetermined treatments, the β-galactosidase staining assay was performed using the senescence β-galactosidase staining kit (9860, Cell Signaling Technology) according to the manufacturer’s instructions. Briefly, cells were fixed in fixation solution for 10 minutes at room temperature, β-galactosidase staining solution was added, and cells were incubated overnight. Stained cells were imaged and counted.

### Immunology multiplex assay.

As described previously (66), CM samples were collected, prepared, and analyzed using a multiplex magnetic bead-based assay (HCYTA-60K MILLIPLEX Human Cytokine/Chemokine/Growth Factor Panel A; Luminex 200 System, Luminex and Multiplex Analysis 5.1 software, Millipore-Sigma) to quantify cytokine concentrations in CM samples.

### WB analysis.

Cells were cultured up to 80% confluence, washed 3 times with PBS, and harvested with 1× cell lysis buffer. The supernatant was mixed with 2× Laemmli buffer (Bio-Rad). The cell lysate was boiled for 10 minutes and loaded onto an SDS-PAGE gel. After transferring to a nitrocellulose membrane (Bio-Rad), the membrane was blocked with 5% milk in PBST buffer and were probed with primary antibodies diluted in 5% BSA or milk in PBST buffer. Horseradish peroxidase–conjugated secondary antibodies were used for detection according to the manufacturer’s instructions and images were obtained using Chemidoc Imaging System (BioRad) and quantified using ImageJ (75).

### Quantitative PCR analysis.

The RNeasy Mini Kit (74106, QIAGEN) was used to isolate total RNA which was reverse transcribed using the qScript cDNA superMix (Quanta Biosciences). The mRNA levels were determined using SYBR Green Real-Time PCR Master Mixes (21203, Bimake) and normalized to ribosomal protein L32 mRNA (RL32, for mRNAs). PCR primers utilized are listed (Supplemental Table 1).

### Dual-luciferase reporter assay.

TGF-β2 promoter luciferase reporter plasmids (800 ng) and pRL-TK (200 ng) were cotransfected into HCC827 DN cells. After 48 hours, cells were treated with DMSO or Osi for an additional 24 hours. Luciferase activity was then measured using the Dual-Luciferase Reporter Assay System (Promega), according to the manufacturer’s instructions.

### Fixed-cell imaging.

Cells were seeded on type I collagen–coated cover glass (no. 1.5) and were fixed using 4% paraformaldehyde for 10 minutes, permeabilized using 0.1% Triton X-100 for 5 minutes, and blocked with 5% BSA for 30 minutes. Overnight incubation was performed primary antibody diluted in blocking buffer at 4°C. Following this, the cells were incubated in Alexa Fluor–conjugated secondary antibodies (1:500) in blocking buffer for 1 hour at room temperature. Nuclei were counterstained and mounted with Prolong Gold Anti-Fade Mountant with DAPI (P35941, Invitrogen). PBS washing was done 3 times after each step and was also used as the solvent in all steps. For surface exposure of VSV-G assay, the cells were not permeabilized.

### Rab6A^+^ vesicle imaging and quantification.

The Rab6A^+^ vesicle imaging was performed by overexpressing WT Rab6A-EGFP expression vector (Addgene). The number of EGFP-Rab6A^+^ vesicles were determined from maximum intensity projections of deconvolved, thresholded (Otsu), and watershed-segmented 3D stacks by particle analysis using ImageJ.

### Golgi structural analysis.

The area occupied by GM130-stained structures was quantified in volume projections with a limiting polygon as described previously (76). The Golgi area was divided by the nucleus area in the same cell to generate a normalized area fraction. Cells were chosen randomly for such measurements. Golgi element size and number were determined from maximum intensity projections of deconvolved, thresholded (Otsu), and watershed-segmented 3D stacks by particle analysis using ImageJ.

### Image acquisition and quantification.

Confocal imaging was performed on an A1+ platform (Nikon Instruments) equipped with 63×/1.4 NA oil, 100×/1.45 NA oil, and 20×/0.75 NA air objectives and 405/488/561 nm laser lines, and images were acquired using NIS-Elements software (Nikon instruments). Routine wide-field fluorescence, bright-field, and phase-contrast imaging were performed on an IX71 microscope (Olympus). The images were processed and fluorescent intensity was analyzed in Fiji/ImageJ. The Golgi organelle area and Golgi element numbers were quantified in volume projections with a limiting polygon through the area occupied by GM130-stained structures as described previously (48).

### FRAP assays.

Cells were cultured overnight in poly-L-lysine–coated 8-well chamber slides (no. 1.5; 155409, Lab-Tek) and Golgi was stained with Cell Light Golgi-GFP BacMam 2.0 overnight (C10592, Thermo Fisher Scientific). FRAP assays were performed as described previously (48), with some modifications. In brief, the recovery was calculated in a circular region of interest of 1 μm diameter. Ten prebleach images were acquired and averaged to obtain an estimated prebleach intensity. Laser power was adjusted to get approximately 80% bleach depth in 2 iterations. Postbleach images were acquired with no delay to record the diffusion component phase and then at 10-second intervals to record the binding component phase. The fluorescence recovery was calculated as described previously (76).

### Immunohistochemistry.

Tissue sections (4 μm) from formalin-fixed, paraffin-embedded lung tumor tissues were stained using an automated immune-stainer platform, the Leica Bond Max automated stainer (Leica Biosystems Nussloch GmbH). Following the Leica Bond protocol, the tissue sections were deparaffinized and rehydrated. Slides were counterstained with hematoxylin, dehydrated, and coverslipped. H&E-stained sections were digitally scanned using the Aperio AT2 slide scanner (Leica Biosystems) under ×20 objective magnification.

### Statistics.

Unless stated otherwise, the results shown are representative of replicated experiments and data represent the mean ± SD from triplicate samples or randomly chosen cells within a field. When comparing EMT scores in human lung cancers, the EMT score was calculated as previously described (77). Kaplan-Meier survival data were generated using KMPlot (78). Statistical evaluations were carried out with Prism 10 (GraphPad Software, Inc.). Unpaired, 2-tailed Student *t* tests were used to compare the means of 2 groups and ANOVA with Dunnett’s test was used to compare multiple groups to a control. *P* values of less than 0.05 were considered statistically significant.

### Study approval.

All mouse studies were approved by the Institutional Animal Care and Use Committee at The University of Texas MD Anderson Cancer Center (Houston, Texas).

### Data availability.

All data associated with this study are present in the manuscript or in the Supplemental figures, tables, and Supporting Data Values file.

## Author contributions

JMK and MG wrote the manuscript. MG conceived, designed, executed, and interpreted the molecular biology, cell biology and in vivo experiments. CW conceived, designed and executed cloning the TGFβ-2 promoter, EGFR sequencing, and assisted MG with Western blot experiments. MN and JVH conceived, designed and executed the generation of OR-cells. ND assisted MG with the Rab6A^+^ vesicle imaging and quantification. AK assisted MG with the FRAP assay and β-galactosidase staining assays and Golgi area quantification. WZ and XL assisted MG with the in vivo experiments. JY bred the mice for the in vivo studies. GYX assisted MG with the VSV-G assay. AC assisted MG with recombinant and neutralizing TGF-β2 experiments. KG assisted MG with Golgi fractionation and vesicle subfractionation studies. WKR directed and interpreted the LC-MS experiments. CJC generated TCGA LUAD cohort EMT-gene signature data. SW conducted quantitative PCR analysis on EMT markers. XT assisted MG with the design, execution, and interpretation of the molecular and cell biology experiments, as well as the manuscript revision. JMK conceived and supervised the project and contributed to the design and interpretation of all experiments.

## Funding support

- NIH grants R01CA236781 and R01CA255021-01 (to JMK), 5R50CA265307 (to MN), and R03CA280382 (to XT).
- Cancer Prevention Research Institute of Texas (CPRIT) grant RP190682 (to WKR; partial support for the UTMB Mass Spectrometry Facility).
- AC was supported by the Cancer Prevention and Research Institute of Texas (CPRIT) Research Training Award CPRIT Training Program (RP210028).

## Supplementary Material

Supplemental data

Unedited blot and gel images

Supporting data values

## Version 1 12/09/2025

In-Press Preview

## Version 2 02/02/2026

Electronic publication

## Version 3 02/10/2026

corrected typo in abstract

## Version 4 02/24/2026

added CPRIT funding support information
