# Supplementary material for: Osimertinib activates a TGF-β2–dependent secretory program that drives lung adenocarcinoma progression
Source: J Clin Invest. 2025 Dec 9;136(3):e198418. doi: 10.1172/JCI198418 (PMC12867136; doi:10.1172/JCI198418)
Supplement: Supplemental data [file jci-136-198418-s008.pdf]

Supplemental figures

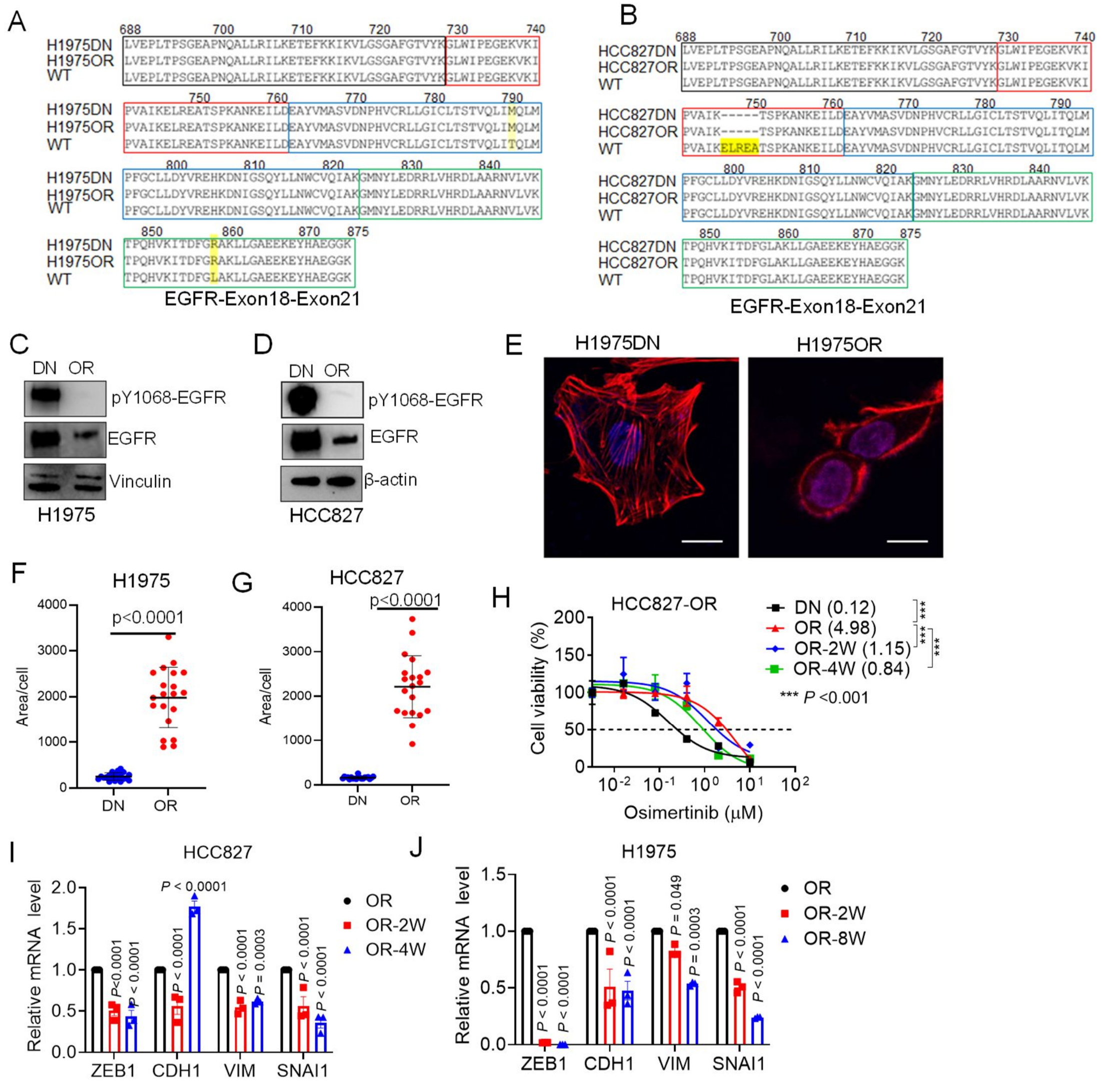

Figure S1. Characterization of drug-naïve (DN) and Osimertinib-resistant (OR) human lung adenocarcinoma (LUAD) cell line pairs. (A, B) Multiple sequence alignment of EGFR exons 18 to 21 in H1975 cells (A) and HCC827 cells (B). DN cells and OR cells were aligned with wild-type (WT) using Jalview software. Exons 18, 19, 20, and 21 are outlined with black, red, blue, and green boxes, respectively. Point mutations in H1975 cells and deletion mutation in HCC827 cells are highlighted in yellow. (C, D) WB analysis of H1975 cells (C) and HCC827 cells (D) demonstrate persistent loss of pY1068 EGFR in OR cells. Vinculin, β-actin loading controls. (E) Confocal micrographs of F-actin (Phalloidin, red) and nucleus (DAPI, blue) in H1975 cells demonstrate distinct morphologies of DN and OR; Scale bars: 10 μm. (F, G) Scatter plot of area per cell (dot) in H1975 cells (F) and HCC827 cells (G). (H) Relative densities of HCC827 DN and OR cells following 3 days of Osimertinib treatment in monolayer culture. Results expressed relative to DMSO control. OR cells were cultured without Osimertinib for 2 and 4 weeks. IC50 values were calculated. (I, J) Quantitative PCR analysis of mRNA levels of EMT markers in HCC827 OR (I) and H1975 OR (J) cells. OR cells were cultured without Osimertinib for 2, 4 or 8 weeks. Data are the mean ± SD from a single experiment incorporating biological replicate samples (n = 3, unless otherwise indicated) and are representative of at least 2 independent experiments. Two-tailed Student's t test (F, G, I, and J). Two-way ANOVA test (H).

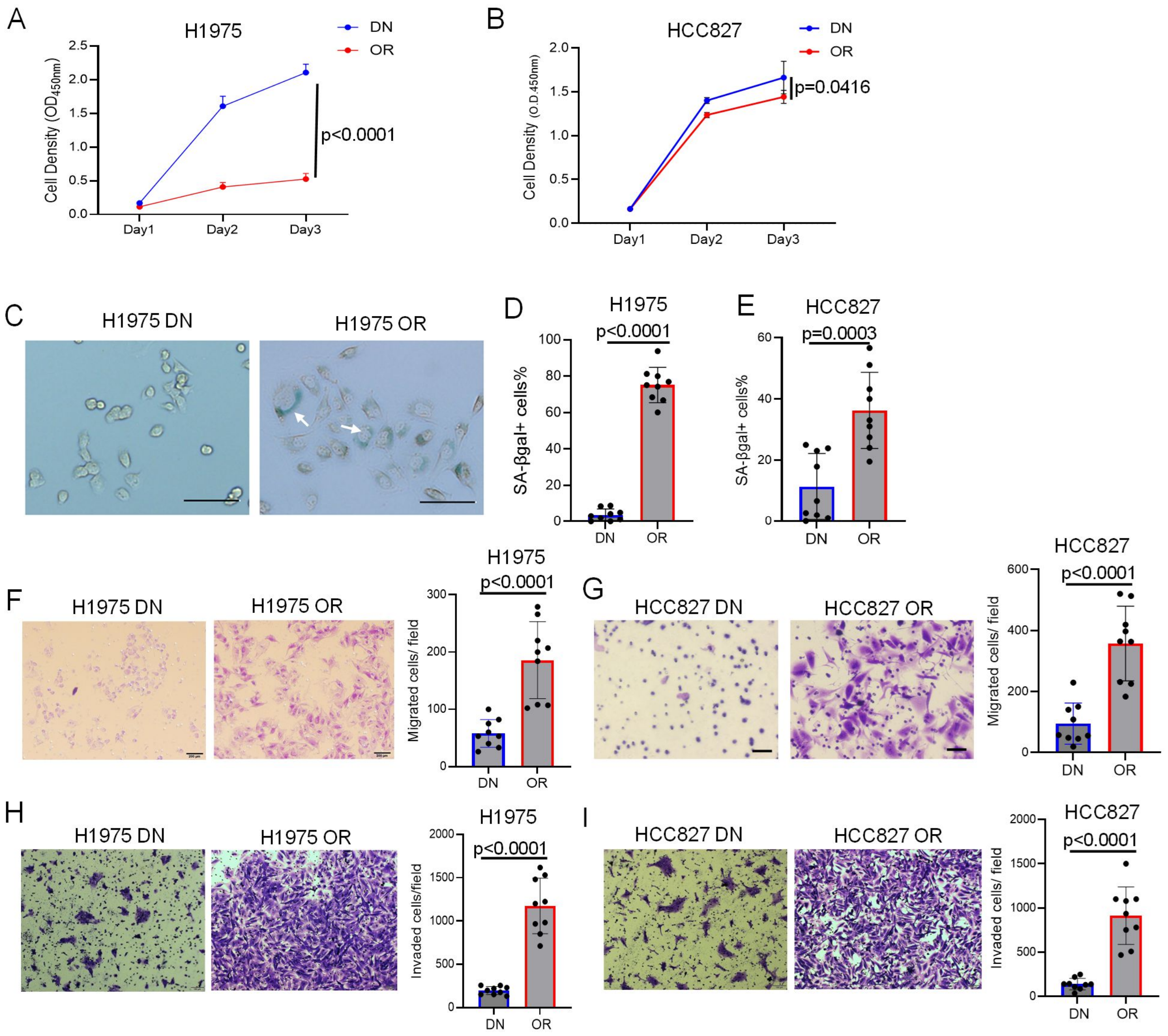

Figure S2. OR cells demonstrate drug-tolerant persistent (DTP) cell features. (A, B) Relative densities of H1975 cells (A) and HCC827 cells (B) (DN and OR) in monolayer culture. (C) SA β-galactosidase staining of H1975 cells. Positive cells are indicated (arrowheads). Scale bars 100 μm. (D, E) Quantification of β-galactosidase^{+} H1975 cells (D) and HCC827 cells (E). Results expressed as a percentage of total cells per field. (F, G) Boyden chamber migration assays on H1975 cells (F) and HCC827 cells (G). Results expressed as an average number of migrated cells per field. Scale bars 200μm. (H, I) Boyden chamber invasion assays on H1975 cells (H) and HCC827 cells (I). Results expressed as an average of invaded cells/field. Scale bars 100μm. Data are the mean ± SD from a single experiment incorporating biological replicate samples (n = 3, unless otherwise indicated) and are representative of at least 2 independent experiments. Two-way ANOVA test (A and B). Two-tailed Student's t test (D-I).

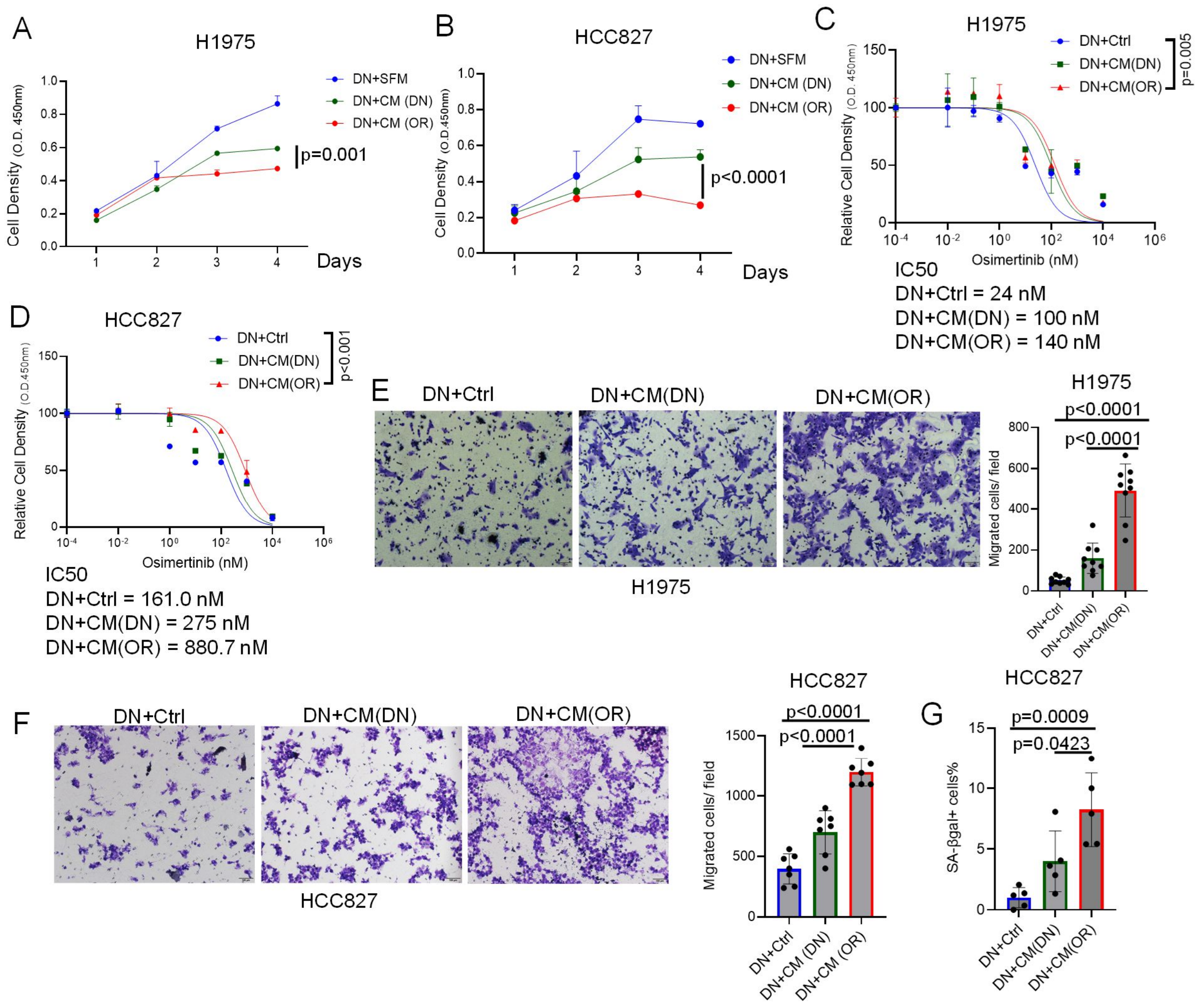

Figure S3. Conditioned medium (CM) transfer experiments. (A) Relative densities of H1975 DN cells treated with OR- or DN-cell-derived CM samples. Control medium (1:1 mixture of Serum-free medium and complete medium) as treatment control. (B) Relative densities of HCC827 DN cells treated with OR- or DN-cell-derived CM samples. SFM treatment control. (C) Relative densities of H1975 DN cells after 72h CM pre-treatment followed by 3 d of Osi treatment. Osi IC50 values (right) were calculated. (D) Relative densities of HCC827 DN cells after 72h of CM sample pre-treatment followed by 3 d of Osi treatment. IC50 values (right) were calculated. (E, F) Boyden chamber migration assays on H1975 DN cells (E) and HCC827 DN cells (F). Cells were pre-treated for 72h with OR- or DN-cell-derived CM samples and seeded into chambers. Migrated cells were visualized and quantified 18 h after seeding. Scale bar, 100μm. Results expressed as an average number of migrated cells per field (bar graphs). (G) HCC827 DN cells were pre-treated for 72h with OR- or DN-cell-derived CM samples and SA β-galactosidase staining assay was performed. Data are the mean ± SD from a single experiment incorporating biological replicate samples (n = 3, unless otherwise indicated) and are representative of at least 2 independent experiments. Two-way ANOVA test (A-D). One-way ANOVA test (E-G).

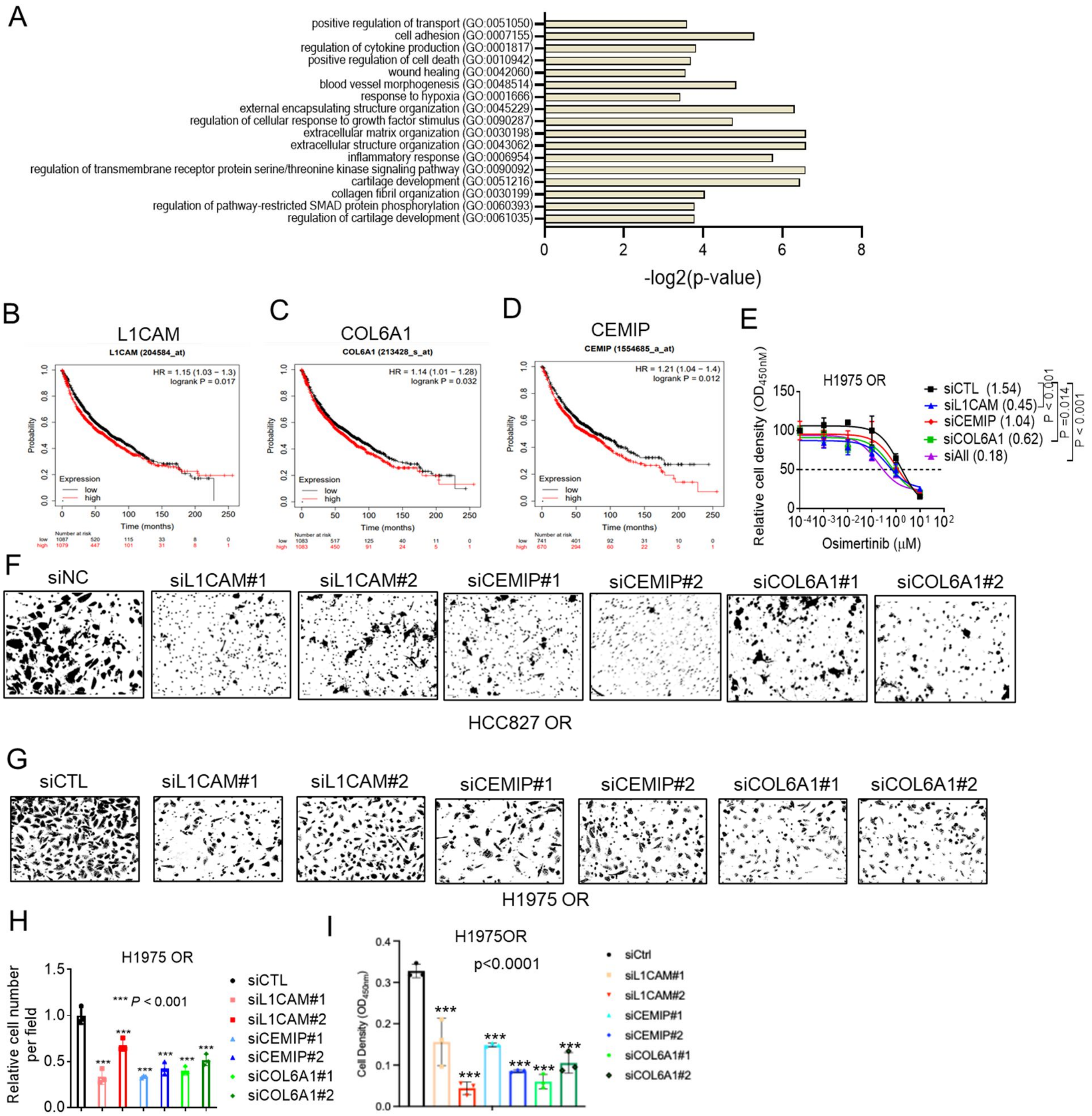

Figure S4. Characterization of the OR cell secretome. (A) Gene Ontology term enrichment analysis of differentially secreted proteins identified by LC-MS analysis of CM samples (H1975 OR versus DN). (B-D) Kaplan-Meier survival analysis of the TCGA LUAD cohorts based on mRNA expression levels of L1CAM (B), COL6A1 (C), or CEMIP (D). Tumors were scored as above (high) or below (low) each gene's median values. (E) Relative densities of siRNA-transfected H1975 OR cells following 3 days of Osimertinib treatment in monolayer culture. Results expressed relative to DMSO control. IC50 values were calculated. (F) Images of HCC827 OR migrated cells in Boyden chambers. (G) Boyden chamber migration assays on siRNA-transfected H1975 OR cells. (H) Results expressed relative to siCtrl. (I) Relative densities of siRNA-transfected HCC827 cells in monolayer culture quantified at 4 d. Data are the mean ± SD from a single experiment incorporating biological replicate samples (n = 3, unless otherwise indicated) and are representative of at least 2 independent experiments. One-sided Fisher's exact test for Gene Ontology analysis. Logrank test (B-D). Two-way ANOVA test (E). One-way ANOVA test (H and I).

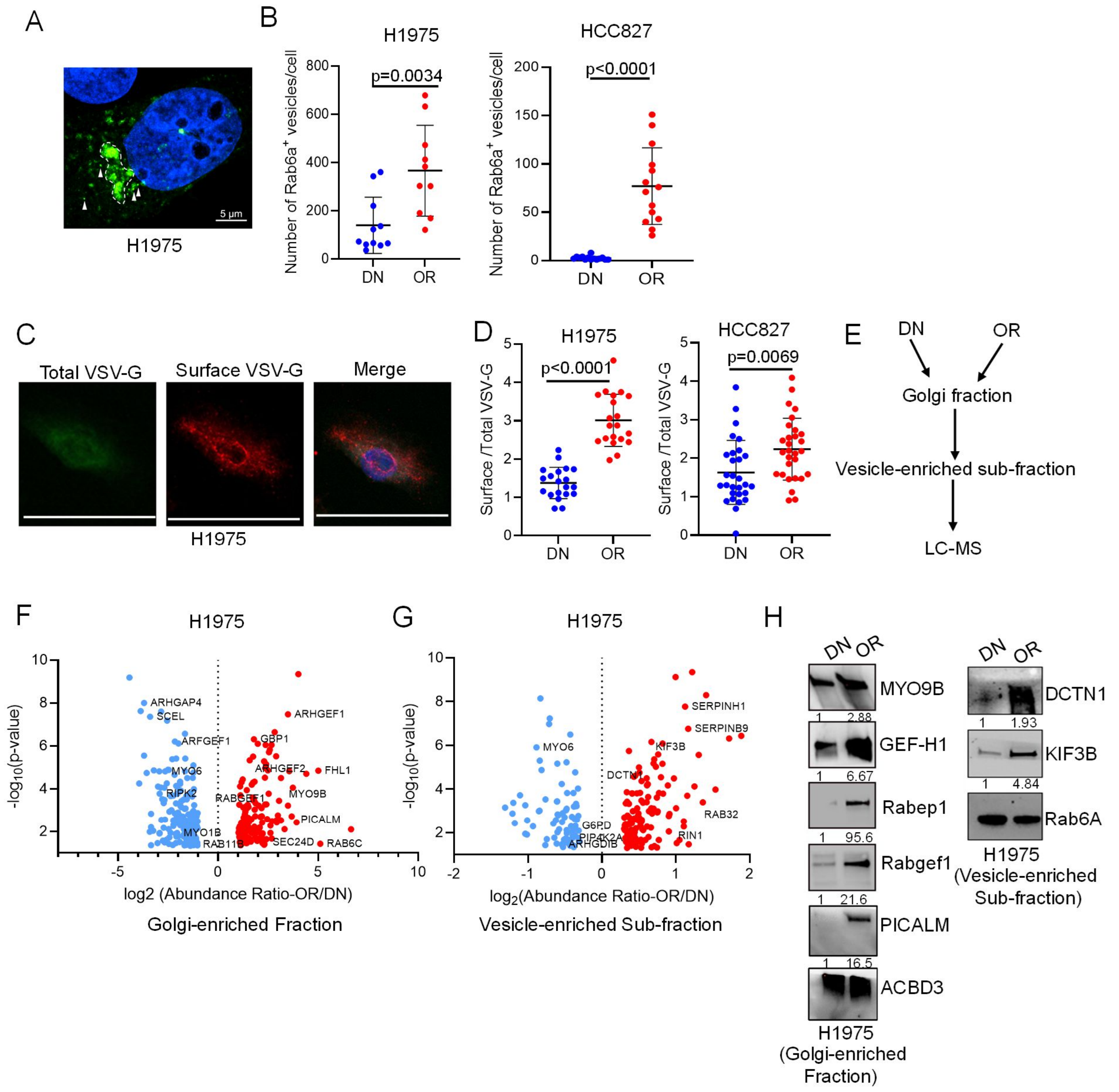

Figure S5. Evidence of conventional secretory pathway activation in OR cells. (A) Confocal micrograph of H1975 DN cells expressing a GFP-tagged Rab6A reporter. Extra-Golgi Rab6A+ vesicles (arrowheads). Golgi delineated by dotted line. Scale bar, 5μm. (B) Extra-Golgi Rab6A+ vesicles per cell (dot) quantified using Graphpad Prism. (C) Confocal micrographs of total and surface VSV-G in H1975 DN cells infected with Ad-EGFP-VSV-G and imaged 30 min after transfer to the permissive temperature (32 °C). Scale bar, 100. (D) Ratio of surface-to-total VSV-G in each cell (dot) after transfer to 32 °C. (E) Schema for LC/MS analysis of Golgi-enriched fractions and secretory vesicle-enriched subfractions from H1975 DN and OR cells. (F, G) Volcano plot of proteins identified by LC-MS analysis of Golgi-enriched fractions (F) and secretory vesicle-enriched subfractions (G) from H1975 cells (DN and OR). Proteins (dots) plotted by P value (y axis) and fold change (OR/DN, x axis). Fold change>2, p <0.05. Proteins of interest are labeled. (H) WB confirmation of selected proteins identified by LC-MS analysis to be present at higher concentrations in OR cells than DN cells. Golgi-enriched fractions (left). Vesicle-enriched subfractions (right). Relative densitometric values under gels. ACBD3 loading control for golgi-enriched fraction. Rab6A loading control for vesicle-enriched subfraction. Data are the mean ± SD from a single experiment and are representative of at least 2 independent experiments. Two-tailed Student's t test (B and D).

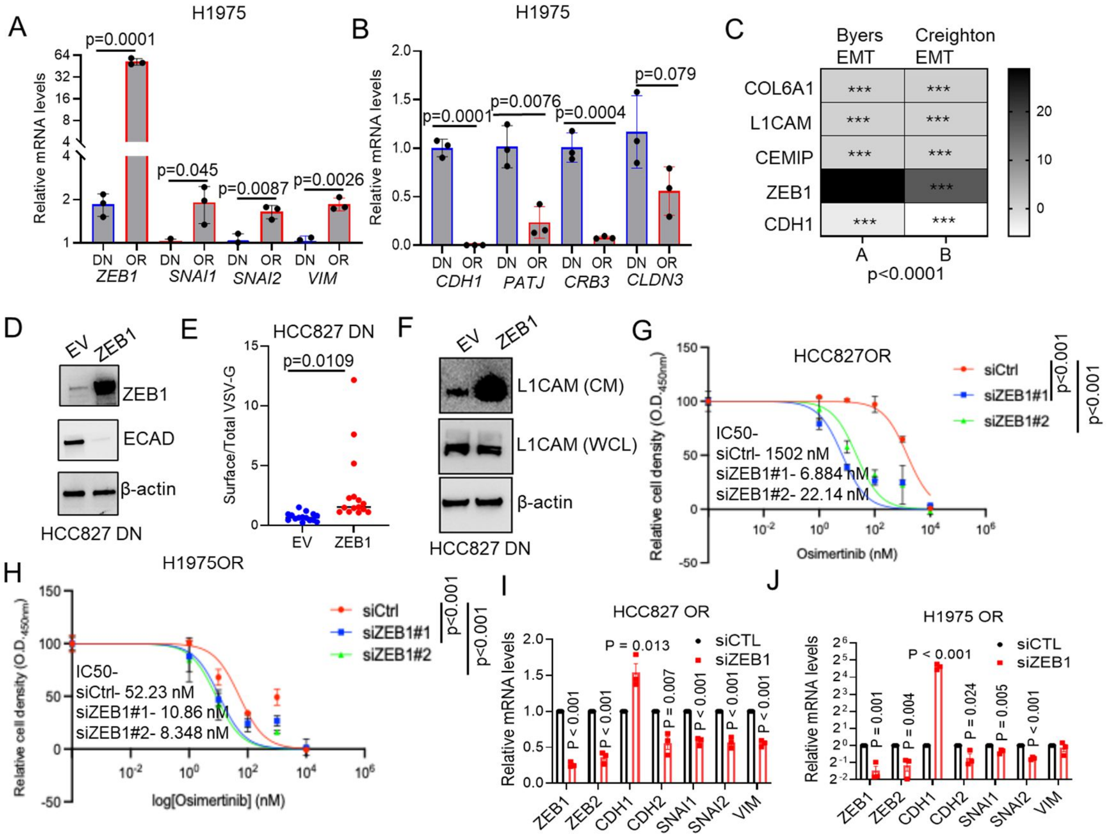

Figure S6. EMT drives secretion in OR cells. (A, B) Quantitative PCR analysis of mRNA levels in H1975 cells (OR and DN). Relative levels of EMT-activating transcription factors (ZEB1, SNA1, SNAI2), VIM, and epithelial polarity complex components (CDH1, PATJ, CRB3, and CLDN3) are indicative of EMT in OR cells. (C) Heat map illustration of correlation between mRNAs levels of secreted proteins and EMT scores (Byers or Creighton) in the TCGA LUAD cohort. r values: Pearson correlation. (D) WB confirmation of ectopic ZEB1 expression in HCC827 DN cells. EV empty vector control. β-actin loading control. (E) Ratio of surface-to-total VSV-G per cell (dot) after transfer to 32 °C. (F) WB analysis of secreted proteins in CM samples following ectopic ZEB1 expression in DN cells. (G) Relative densities of siRNA-transfected HCC827 OR cells following 3 days of Osimertinib treatment in monolayer culture. Results expressed relative to DMSO control. IC50 values were calculated. (H) Relative densities of siRNA-transfected H1975 OR cells following 3 days of Osimertinib treatment in monolayer culture. Results expressed relative to DMSO control. IC50 values were calculated (I) Quantitative PCR analysis of mRNA levels in siRNA-transfected HCC827 OR cells. Relative levels of EMT-activating transcription factors ZEB1, ZEB2, SNA1, SNAI2, VIM, and epithelial marker CDH1 are indicative of EMT in OR cells. (J) Quantitative PCR analysis of mRNA levels in siRNA-transfected H1975 OR cells. Relative levels of EMT-activating transcription factors ZEB1, ZEB2, SNA1, SNAI2, VIM, and epithelial marker CDH1 are indicative of EMT in OR cells. Data are the mean ± SD from a single experiment incorporating biological replicate samples (n = 3, unless otherwise indicated) and are representative of at least 2 independent experiments. Two-tailed Student's t test (A, B, E, I, and J). Two-way ANOVA test (G and H).

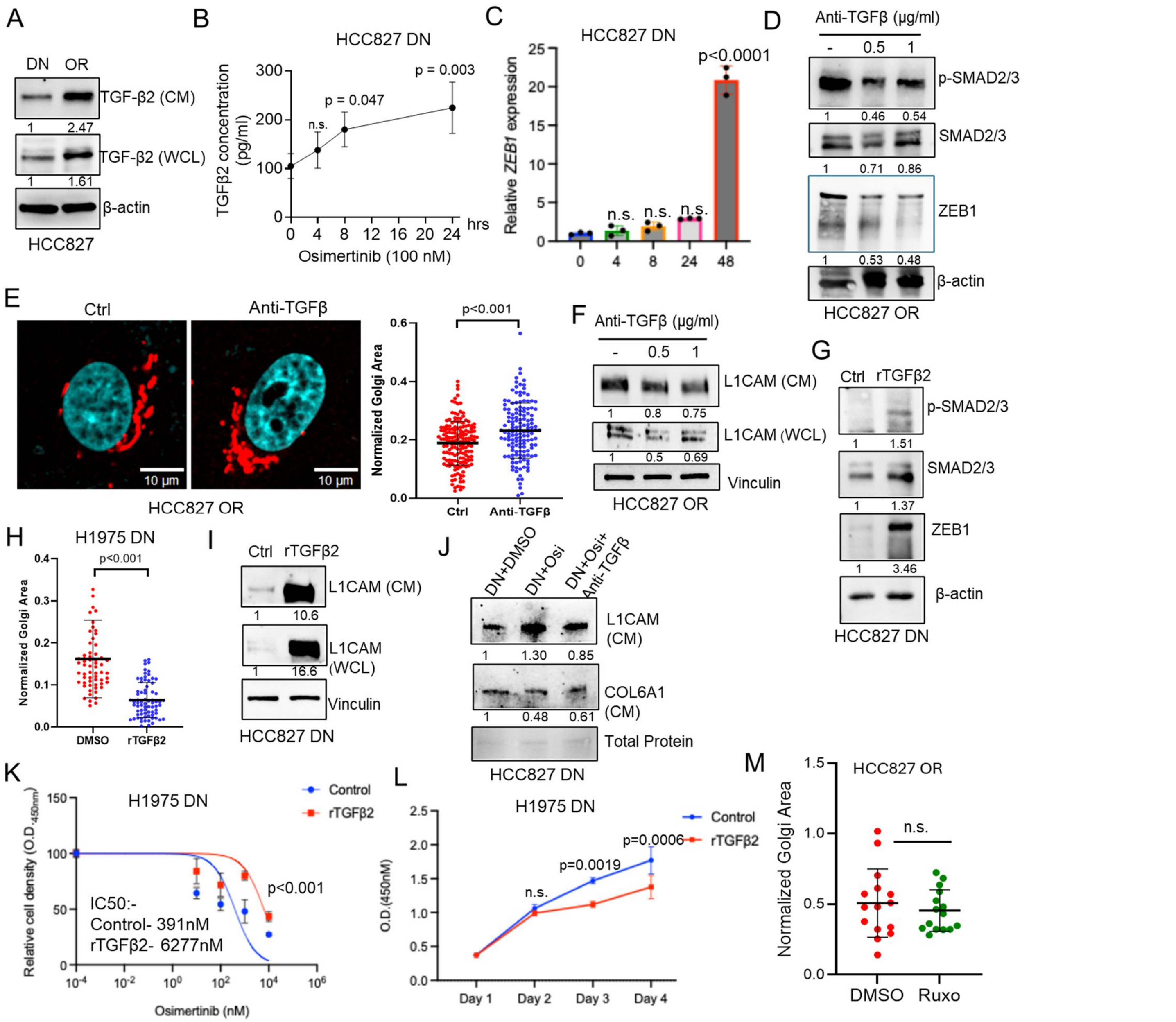

Figure S7. Enhanced TGFβ2-secretion upon Osimertinib treatment induces an EMT-dependent secretory state. (A) TGFβ2 WB analysis of CM and WCL samples from HCC827 cells (DN or OR). (B) Enzyme-linked immunosorbent assays of CM samples show that increased TGFβ2 secretion is detectable 4 h after initiating Osimertinib treatment in HCC827 DN cells. (C) Quantitative PCR analysis shows that ZEB1 mRNA levels are upregulated 24 h after initiating Osimertinib treatment in HCC827 DN cells. (D) WB confirmation of SMAD2/3 dephosphorylation by 0.5 and1 ug/ml neutralizing anti-TGFβ antibody treatment of HCC827 OR cells. β-actin loading control. (E) Confocal micrographs of anti-GM130 antibody-stained HCC827 OR cells treated with or without anti-TGFβ. Golgi (red). Nuclei (DAPI, cyan). Scale bars: 10 μm. Scatter plot: Golgi areas following TGFβ neutralization in HCC827 OR cells (dots). Values normalized based on nuclear areas. (F) WB analysis of L1CAM in CM and WCL samples following TGFβ neutralization in HCC827 OR cells. (G) WB confirmation of increased SMAD2/3 phosphorylation by 100nM recombinant TGFβ2 treatment of HCC827 DN cells. β-actin loading control. (H) Scatter plot of Golgi areas in HCC827 DN cells (dots) following treatment with 100 nM recombinant TGFβ2. Values normalized based on nuclear areas. (I) WB analysis of L1CAM in CM and WCL samples following recombinant TGFβ2 treatment of HCC827 DN cells. (J) WB analysis of L1CAM and COL6A1 in CM samples from HCC827 DN cells treated for 24 h with DMSO, 100nM Osimertinib or Osimertinib in combination with 1ug/ml neutralizing anti-TGFβ antibody. Ponceau-stained gel included as loading control. (K) Relative densities of H1975 DN cells pre-treated with 100nM recombinant TGFβ2 and following 3 days of Osimertinib treatment in monolayer culture. Results expressed relative to DMSO control. IC50 values were calculated. (L) Relative

densities of H1975 DN cells treated with 100nM recombinant TGFβ2. (M) Scatter plot of Golgi areas in HCC827 OR cells (dots) following 24 h of 1μM Ruxo treatment. Values normalized based on nuclear areas. Data are the mean ± SD from a single experiment incorporating biological replicate samples (n = 3, unless otherwise indicated) and are representative of at least 2 independent experiments. One-way ANOVA test (B and C). Two-tailed Student's t test (E, H, and M). Two-way ANOVA test (K and L).

Table S1. Quantitative-PCR primers.

Gene	Forward Primer	Reverse Primer
L1CAM	ACGAGGGATGGTGTCCACTTCAAA	TTATTGCTGGCAAAGCAGCGGTAG
COL6A1	AGCTCAATGTCATTTCTTGC	AGGTGTAATCTGGACACTTC
CEMIP	ACCGAGCACATTCCAACTACCG	GGCAGAGATGATTGAGAGGAACG
ZEB1	GCACCTGAAGAGGACCAGAG	ATGGGGGCGTTGTCATTCAC
CDH1	GCCTCCTGAAAAGAGAGTGGAAG	TGGCAGTGTCTCTCCAAATCCG
PATJ	TAAGCGCCATAGCAGCTCAG	CAGCTGCTCCTCTGTGCTT
CRB3	TTAAGCGCTCCCCATCCAAG	GACGCACGGGGTATGTATGT
CLDN3	GCCACCAAGGTCGTCTACTC	CCTGCGTCTGTCCCTTAGAC
VIM	AGGCAAAGCAGGAGTCCACTGA	ATCTGGCGTTCCAGGGACTCAT
SNAI1	TGCCCTCAAGATGCACATCCGA	GGGACAGGAGAAGGGCTTCTC
SNAI2	ATCTGCGGCAAGGCGTTTTCCA	GAGCCCTCAGATTTGACCTGTC
TWIST1	GCCAGGTACATCGACTTCCTCT	TCCATCCTCCAGACCGAGAAGG
RPL32	ACAAAGCACATGCTGCCCAGTG	TTCCACGATGGCTTTGCGGTTC
