# Supplementary material for: Osimertinib activates a TGF-β2–dependent secretory program that drives lung adenocarcinoma progression
Source: J Clin Invest. 2025 Dec 9;136(3):e198418. doi: 10.1172/JCI198418 (PMC12867136; doi:10.1172/JCI198418)

Unedited blot for Fig 1. F

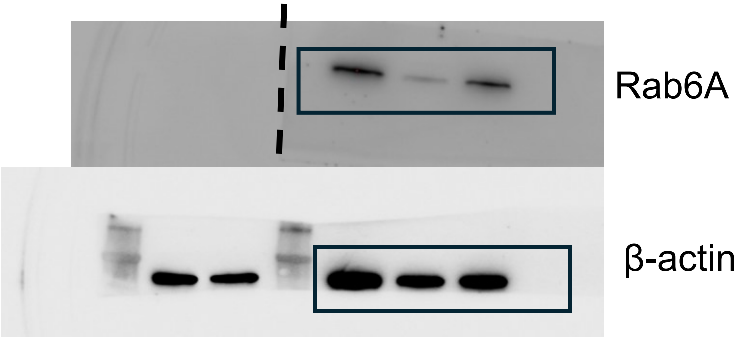

Unedited blot/gel for Fig. 3B

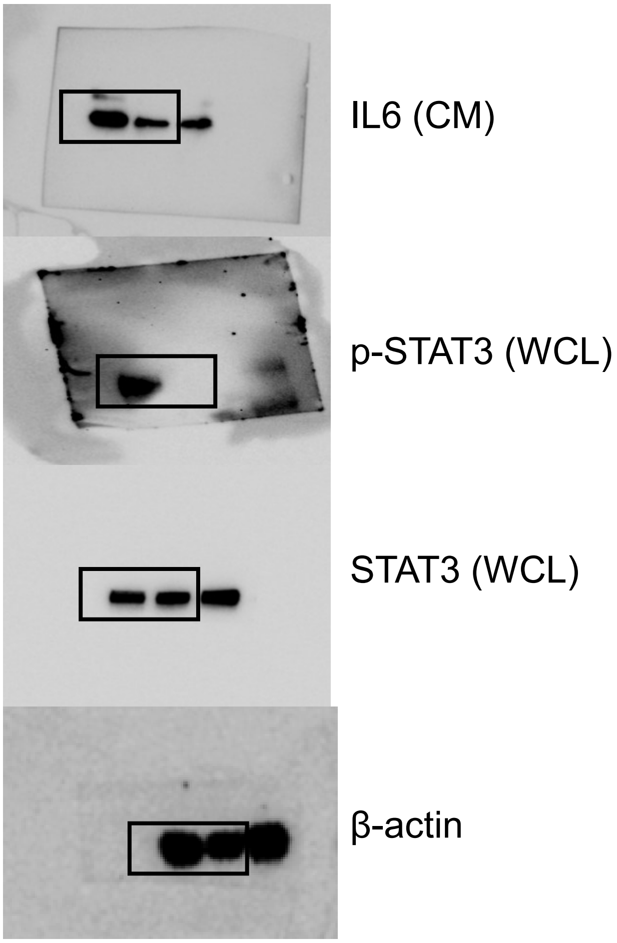

Unedited blot/gel for Fig. 3F

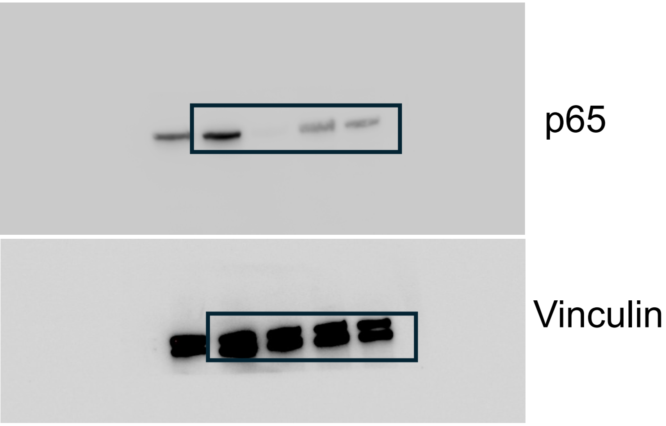

Unedited blot for Fig. 4B WCL

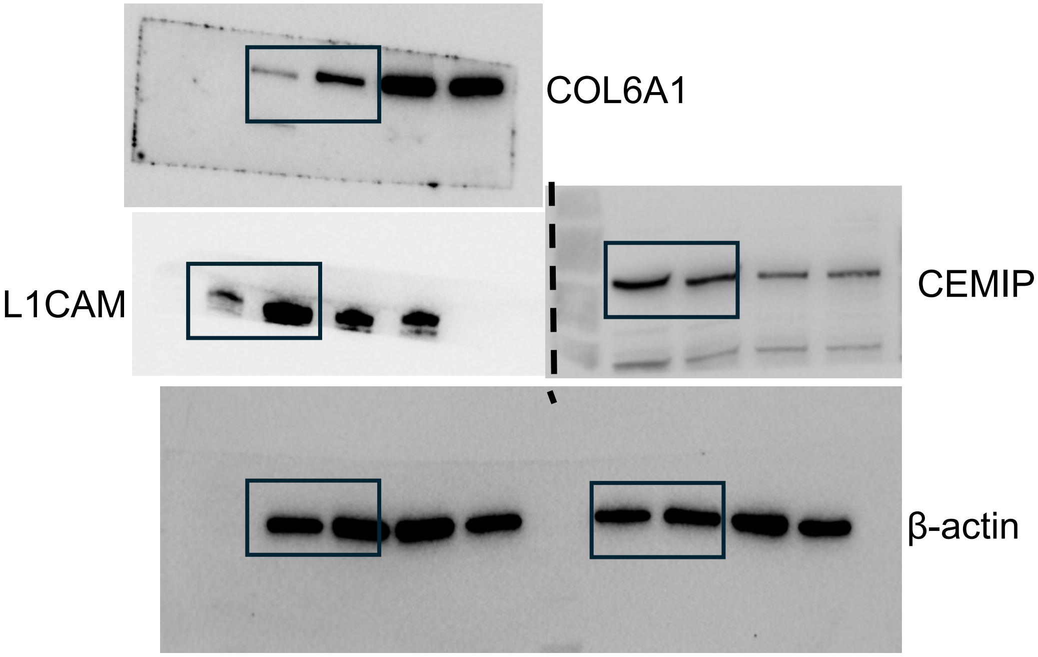

Unedited blot for Fig. 4B CM

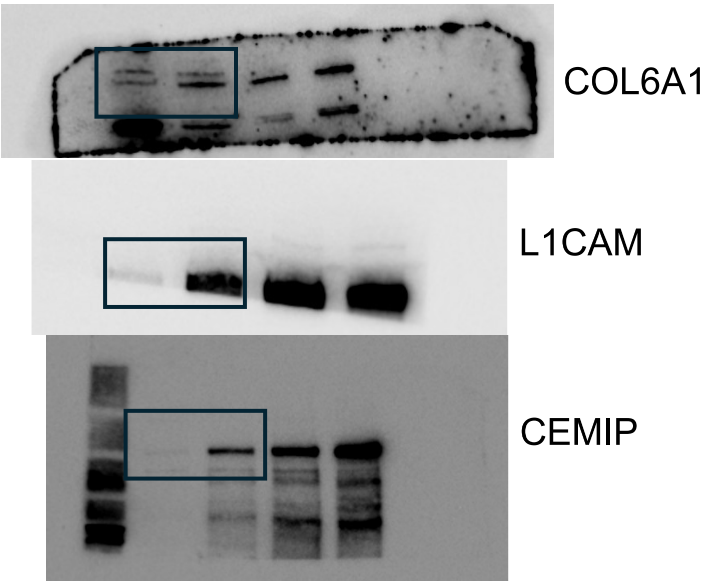

Unedited blot for Fig. 4C WCL

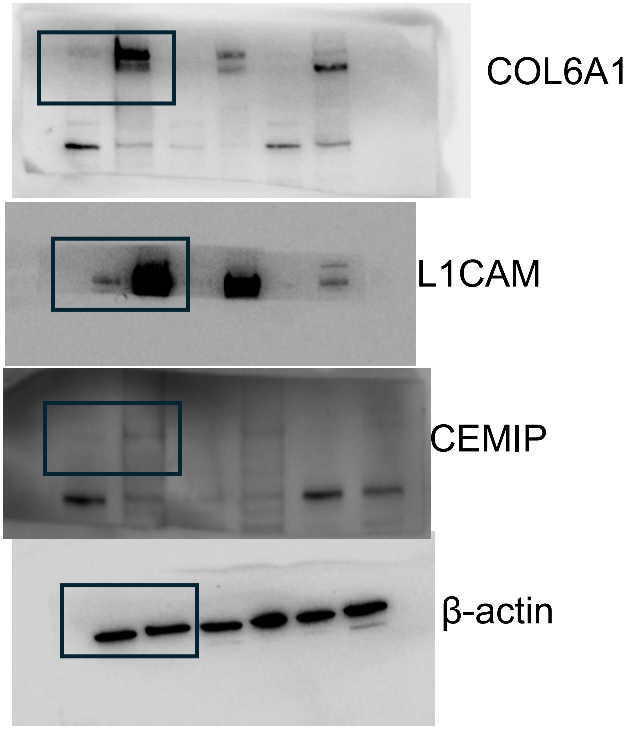

Unedited blot for Fig. 4C CM

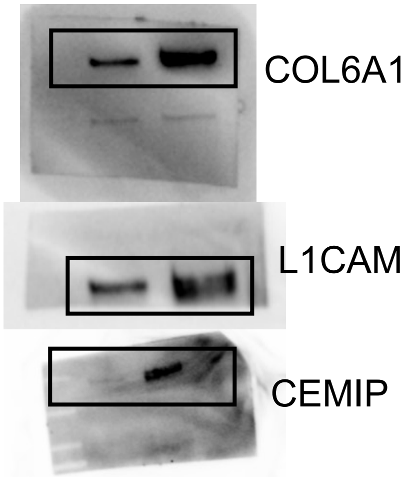

Unedited blot for Fig. 4D CEMIP KD

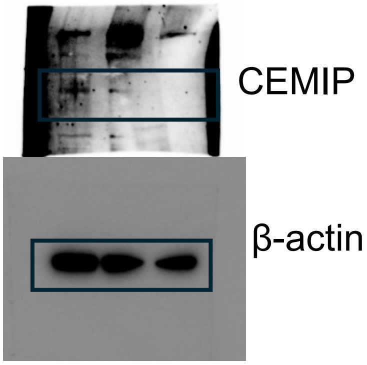

Unedited blot for Fig. 4D COL6A1 KD

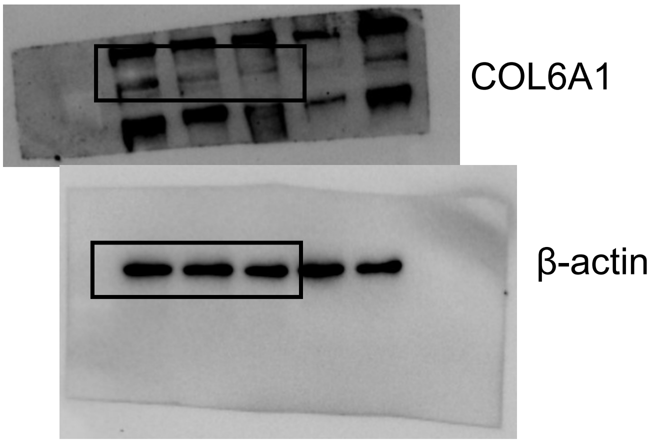

Unedited blot for Fig. 4D L1CAM KD

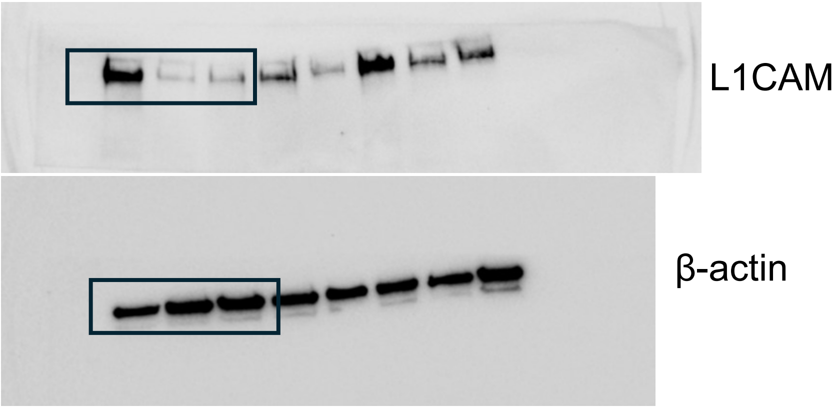

Unedited blot for Fig. 4H

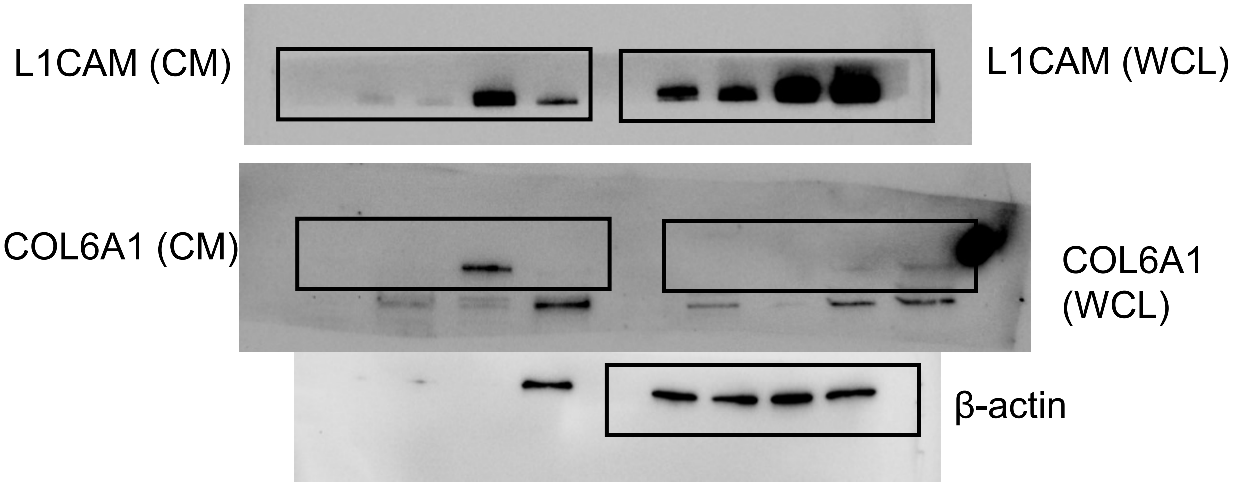

Unedited blot for Fig. 4I

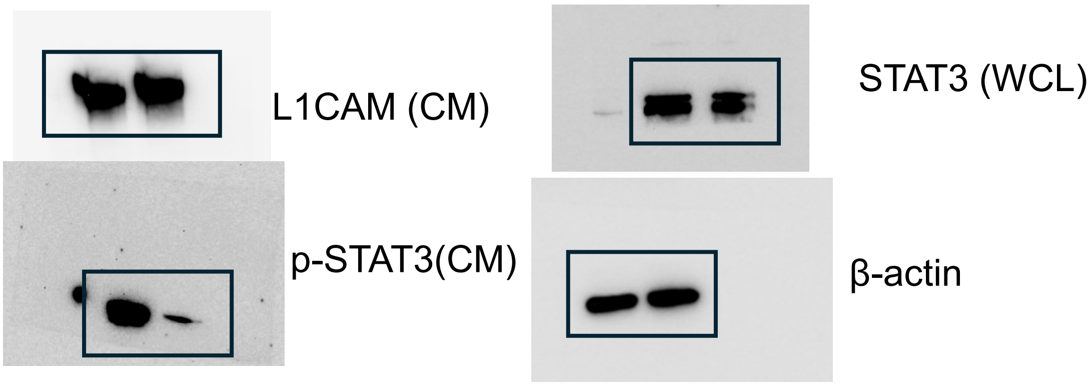

Unedited blot for Fig. 6C

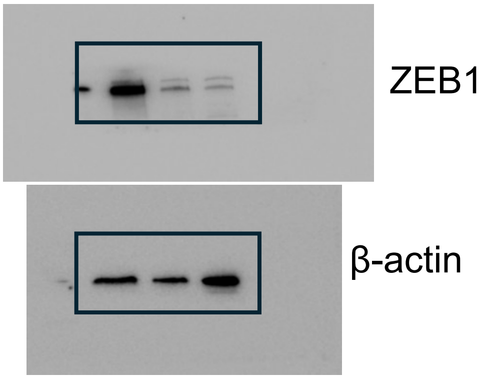

Unedited blot for Fig. 6 I

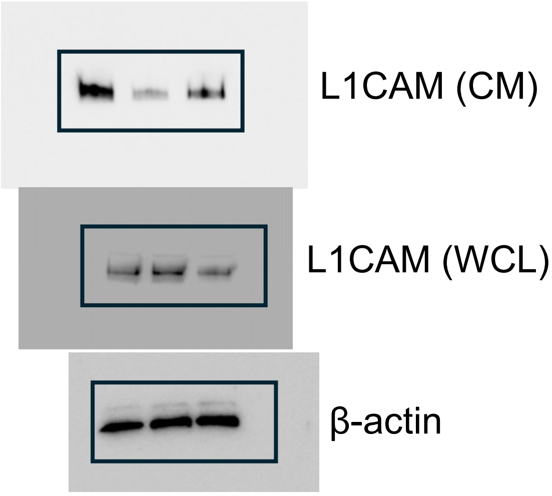

Unedited blot for Fig. 6J

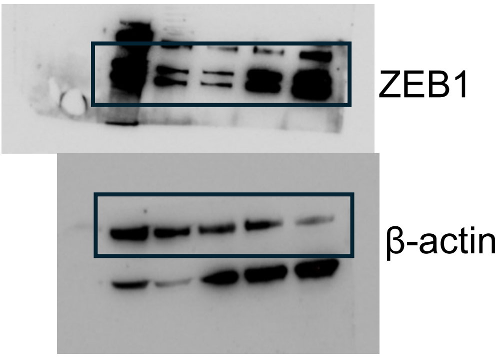

Unedited blot for Fig. 7A

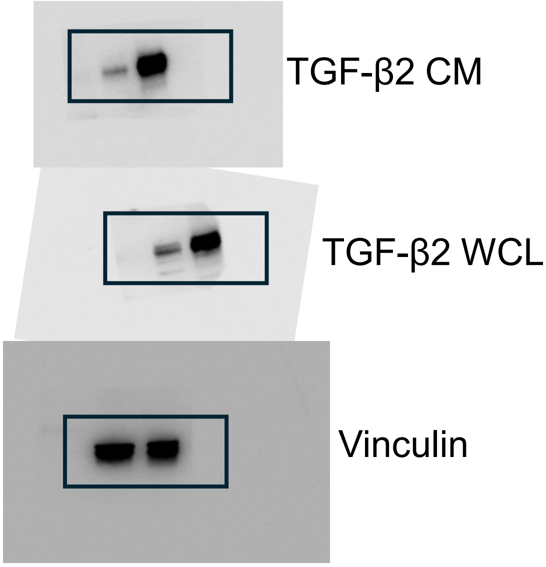

Unedited blot for Fig. 7E

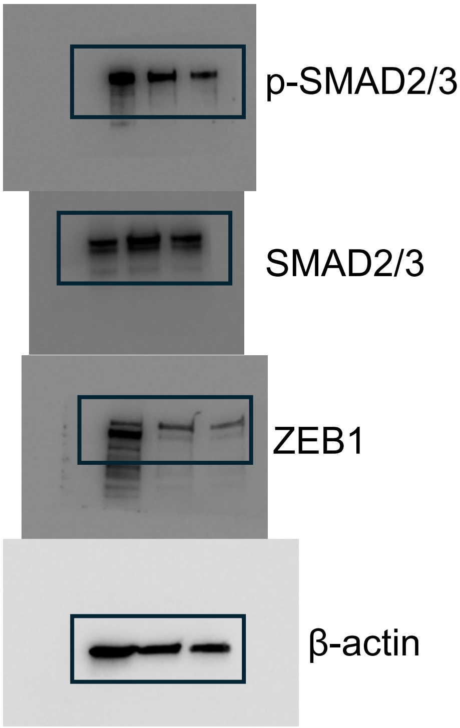

Unedited blot for Fig. 7G

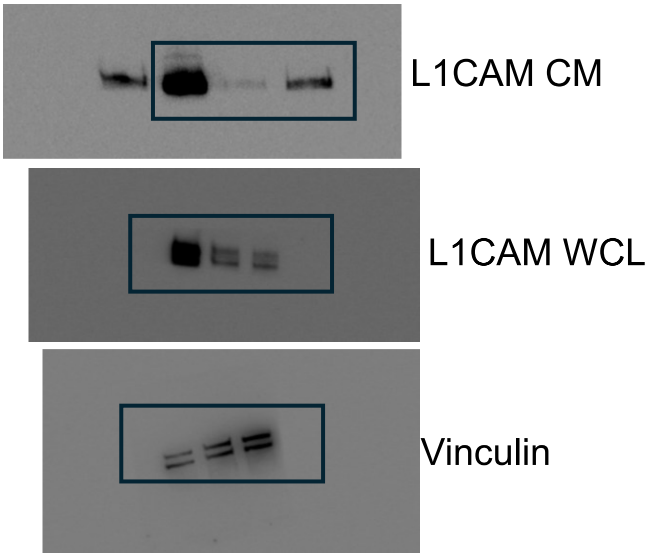

Unedited blot for Fig. 7H

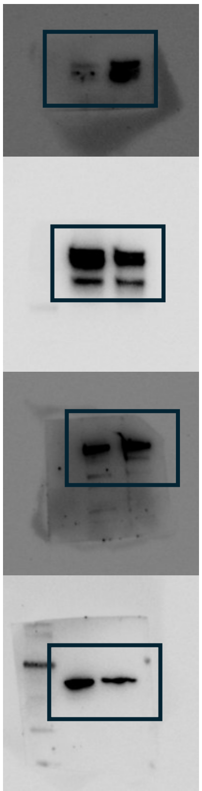

p-SMAD2/3

SMAD2/3

ZEB1

β-actin

Unedited blot for Fig. 7K

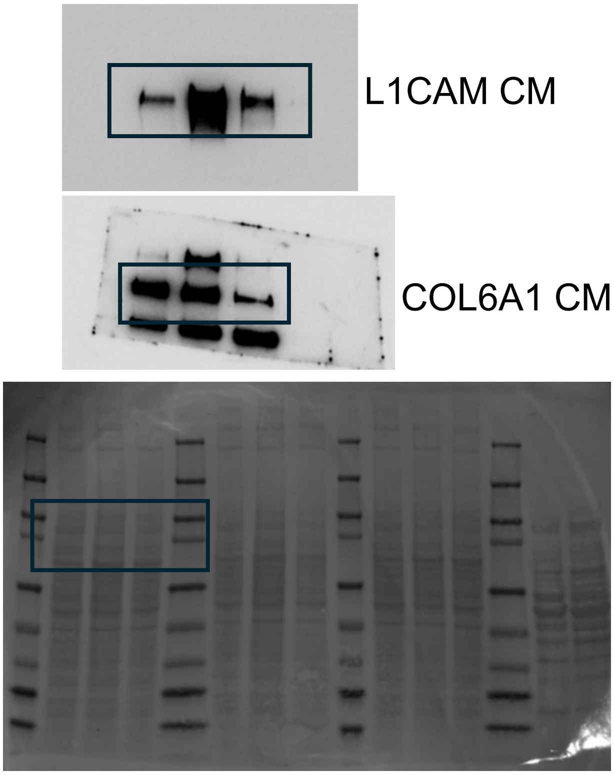

L1CAM CM

COL6A1 CM

Unedited blot for Fig. 7J

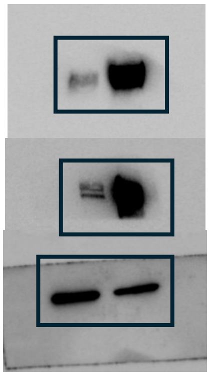

L1CAM CM

L1CAM WCL

β-actin

Unedited blot for Fig S1. C

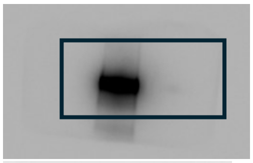

pY1068-EGFR

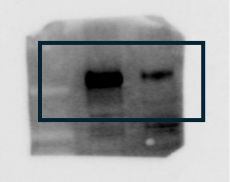

EGFR

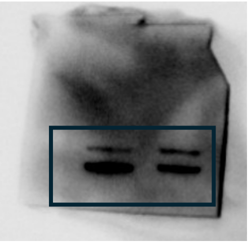

Vinculin

Unedited blot for Fig S1. D

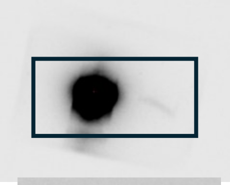

pY1068-EGFR

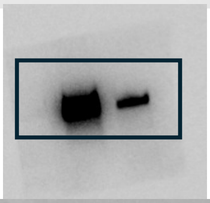

EGFR

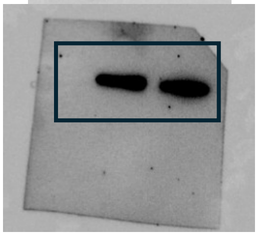

β-actin

Unedited blot for Fig. S5H

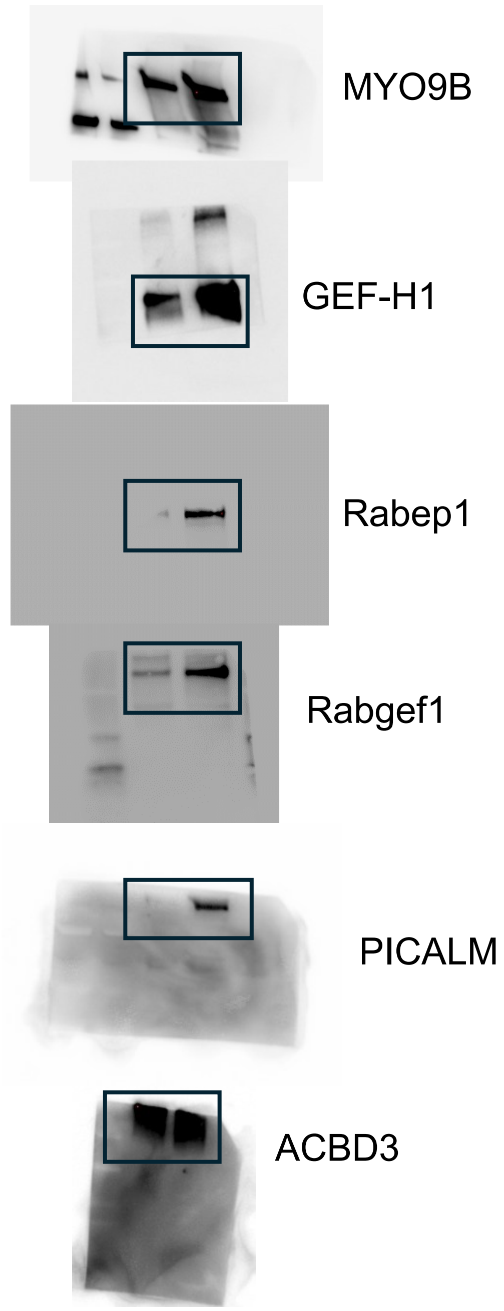

Unedited blot for Fig. S5H

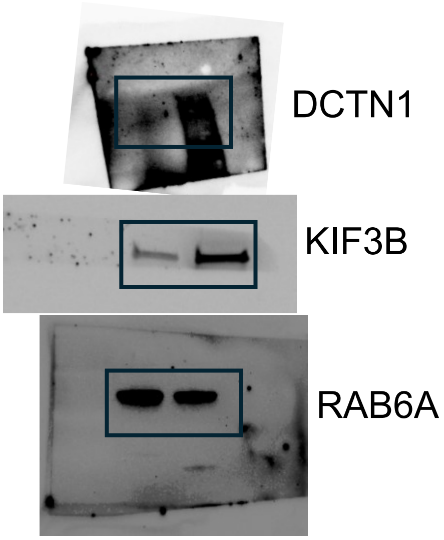

Unedited blot for Fig S6. D

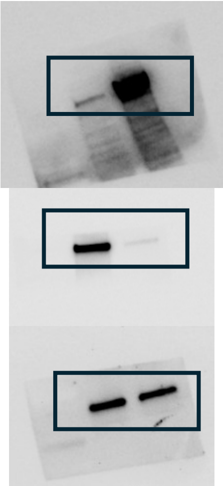

Unedited blot for Fig S6. F

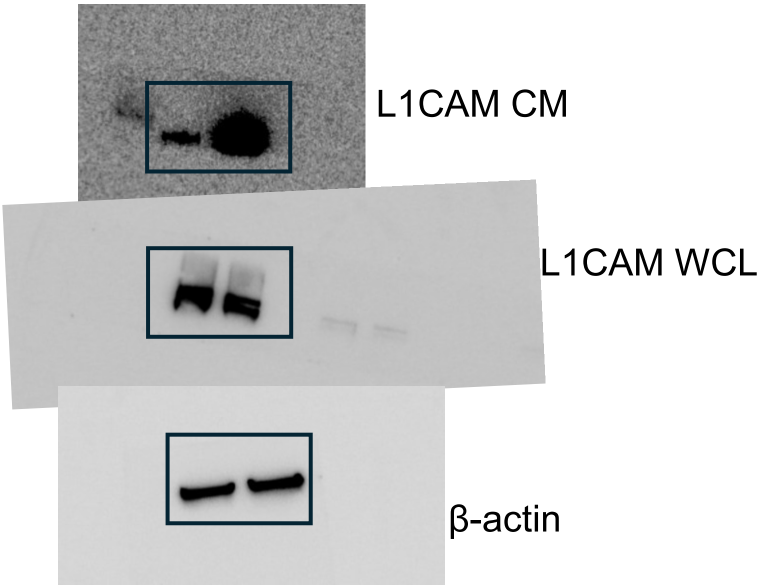

Unedited blot/gel for Fig. S7 A

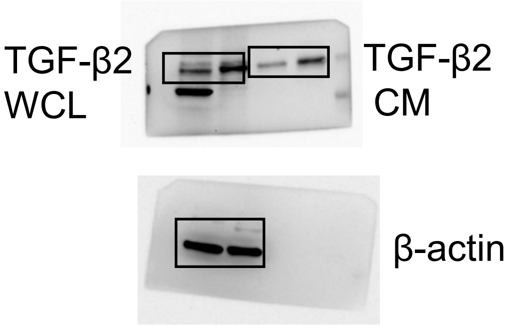

Unedited blot/gel for Fig. S7 D

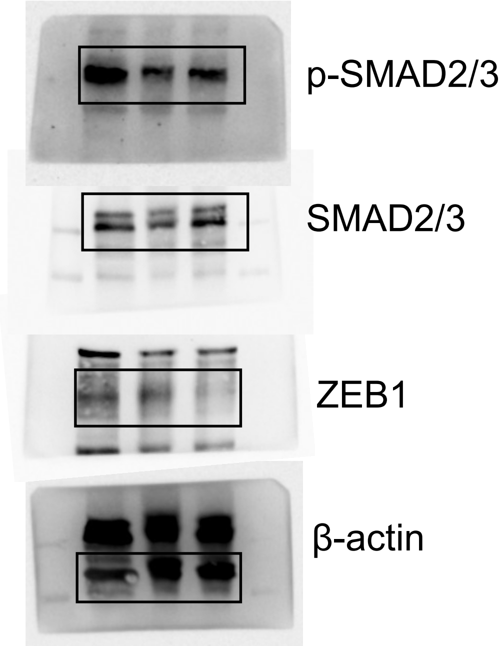

Unedited blot/gel for Fig. S7 F

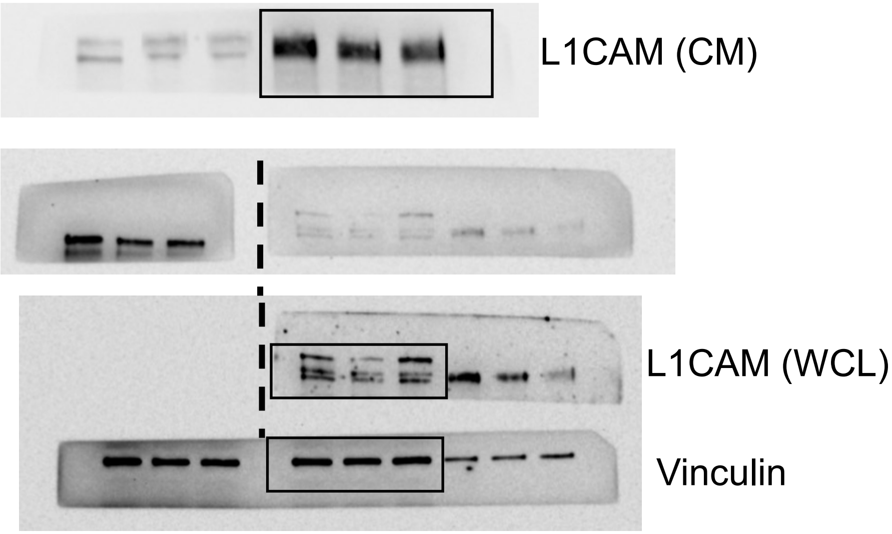

Unedited blot/gel for Fig. S7 G

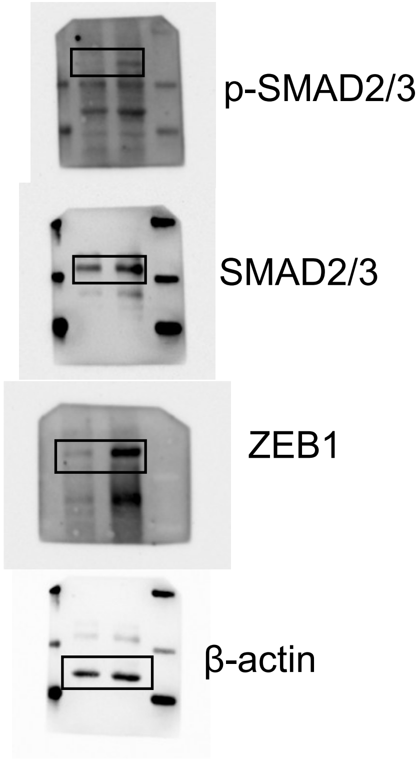

Unedited blot/gel for Fig. S7 I

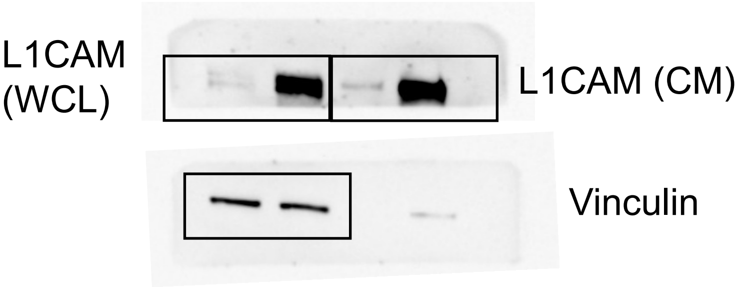

Unedited blot/gel for Fig. S7 J

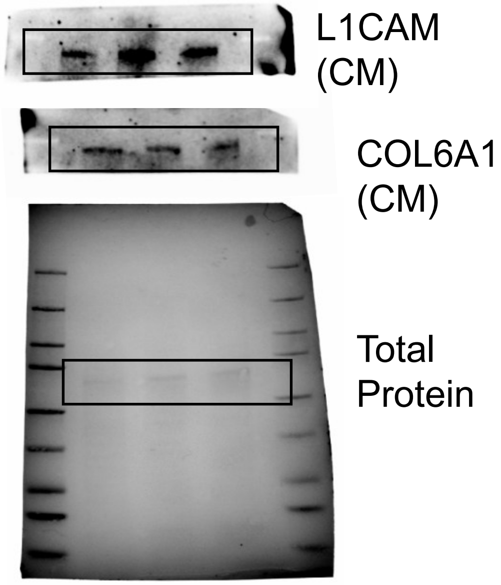

Supplement: Unedited blot and gel images [file jci-136-198418-s009.pdf]
